# Targeted BRAF Inhibition Impacts Survival in Melanoma Patients with High Levels of Wnt/β-Catenin Signaling

**DOI:** 10.1371/journal.pone.0094748

**Published:** 2014-04-14

**Authors:** Andy J. Chien, Lauren E. Haydu, Travis L. Biechele, Rima M. Kulikauskas, Helen Rizos, Richard F. Kefford, Richard A. Scolyer, Randall T. Moon, Georgina V. Long

**Affiliations:** 1 Division of Dermatology, University of Washington Department of Medicine, Seattle, Washington, United States of America; 2 The Group Health Research Institute, Seattle, Washington, United States of America; 3 Melanoma Institute of Australia, Sydney, New South Wales, Australia; 4 The University of Sydney, Sydney, New South Wales, Australia; 5 Westmead Institute for Cancer Research, Westmead Millennium Institute, Westmead, New South Wales, Australia; 6 Westmead Hospital, Sydney, New South Wales, Australia; 7 Royal Prince Alfred Hospital, Sydney, New South Wales, Australia; 8 The Howard Hughes Medical Institute, Chevy Chase, Maryland, United States of America; University of Connecticut Health Center, United States of America

## Abstract

Unprecedented clinical responses have been reported in advanced stage metastatic melanoma patients treated with targeted inhibitors of constitutively activated mutant BRAF, which is present in approximately half of all melanomas. We and others have previously observed an association of elevated nuclear β-catenin with improved survival in molecularly-unselected melanoma patients. This study sought to determine whether levels of Wnt/β-catenin signaling in melanoma tumors prior to treatment might predict patient responses to BRAF inhibitors (BRAFi). We performed automated quantification of β-catenin immunohistochemical expression in pretreatment *BRAF*-mutant tumors from 32 BRAFi-treated melanoma patients. Unexpectedly, patients with higher nuclear β-catenin in their tumors did not exhibit the survival advantage previously observed in molecularly-unselected melanoma patients who did not receive BRAFi. In cultured melanoma cells treated with long-term BRAFi, activation of Wnt/β-catenin signaling is markedly inhibited, coinciding with a loss of the enhancement of BRAFi-induced apoptosis by WNT3A observed in BRAFi-naïve cells. Together, these observations suggest that long-term treatment with BRAFi can impact the interaction between BRAF/MAPK and Wnt/β-catenin signaling to affect patient outcomes. Studies with larger patient cohorts are required to determine whether nuclear β-catenin expression correlates with clinical responses to BRAFi and to specific mechanisms of acquired resistance to BRAFi. Understanding these pathway interactions will be necessary to facilitate efforts to individualize therapies for melanoma patients.

## Introduction

The incidence and mortality associated with melanoma has risen steadily since the 1970s in the USA, Europe and Australia [1], and the five-year survival rate of 5–15% for patients with advanced stage metastatic disease has remained stagnant over that time. Approximately half of all melanoma tumors harbor activating mutations in *BRAF*, with *BRAF^V600E^* and *BRAF^V600K^* representing approximately 70–90% and 10–30% of mutations, respectively [2]–[10]. Mutation-targeted BRAF inhibitors (BRAFi) such as vemurafenib (PLX4032) and dabrafenib (GSK2118436) represent a landmark development in the treatment of advanced stage BRAF*^V600E/K^*-mutant metastatic melanoma, with objective response rates of approximately 50%, and in phase III trials, a significant improvement in progression-free survival (PFS) and overall survival (OS) compared with dacarbazine chemotherapy [10]–[14]. In addition, almost all patients with tumors harboring activating *BRAF* mutations in these trials exhibit some degree of tumor reduction, even if they do not meet the criteria for an objective clinical response.

Despite the promise of these targeted BRAFi, most patients develop recurrence and relapse at a median of 6–7 months. Studies utilizing patient tumor samples and preclinical models have identified several pathways to the development of BRAFi resistance, and the majority of resistance mechanisms identified to date appear to result in reactivation of the MAP kinase (MAPK) pathway as demonstrated by high levels of phosphorylated ERK1/2 [15]–[20]. Clinically, several questions remain unanswered. For example, what types of molecular and cellular determinants underlie the heterogeneity in therapeutic responses observed across patients with tumors harboring activating *BRAF* mutations and how can these determinants be utilized to predict clinical responses and tailor therapies? Such determinants may be utilized to develop molecular assays that facilitate the identification or selection of optimized drug combinations for patients.

The Wnt/β-catenin signaling pathway has been implicated as an important regulator of melanoma despite the fact that activating mutations in core pathway members appear to be rare in this disease. This signaling pathway is activated by secreted ligands including WNT3A, which is the WNT isoform most often used for activating Wnt/β-catenin signaling in laboratory studies. Frequently, the activation of Wnt/β-catenin signaling has been detected through the measurement of endogenous downstream target genes such as *AXIN2*, which encodes a core pathway protein that promotes the degradation of β-catenin [21]. In patient tissue samples, another surrogate marker of activated Wnt/β-catenin signaling is the immunohistochemical detection of cytoplasmic or nuclear β-catenin, which accumulates in cells upon activation of the pathway [21]. Multiple studies have observed that loss of nuclear or cytoplasmic β-catenin, the downstream effector protein of Wnt, is associated with disease progression and decreased survival in patients with melanoma [22]–[26]. Wnt/β-catenin signaling in melanoma cells is negatively regulated by BRAF^V600E^ [27]. However, Wnt/β-catenin signaling also reciprocally regulates BRAF-mediated signaling. In *BRAF*-mutant cell lines, the activation of Wnt/β-catenin signaling in combination with BRAFi synergistically enhanced apoptosis *in vitro* and increased inhibition of tumor growth *in vivo* [27]. Furthermore, melanoma cell apoptosis mediated by BRAFi unexpectedly required β-catenin and intact Wnt/β-catenin signaling [27].

Given that elevated Wnt/β-catenin signaling has been associated with improved melanoma survival outcomes in molecularly unselected patients along with enhancement of apoptosis with BRAFi in laboratory melanoma models, we hypothesized that higher levels of Wnt/β-catenin signaling in pre-treatment melanoma tumors (as measured by increased nuclear β-catenin) might predict a better clinical response to BRAFi. To address this hypothesis, we performed a retrospective analysis of Wnt/β-catenin signaling of pretreatment melanoma specimens from patients treated clinically with BRAFi for metastatic melanoma. In parallel, we studied the effects of long-term BRAFi treatment in cultured melanoma cells. Our results extend the previous model for how Wnt/β-catenin and BRAF/MAPK signaling interact in melanoma.

## Results

### Patient characteristics and measurement of β-catenin

**Figure 1:**
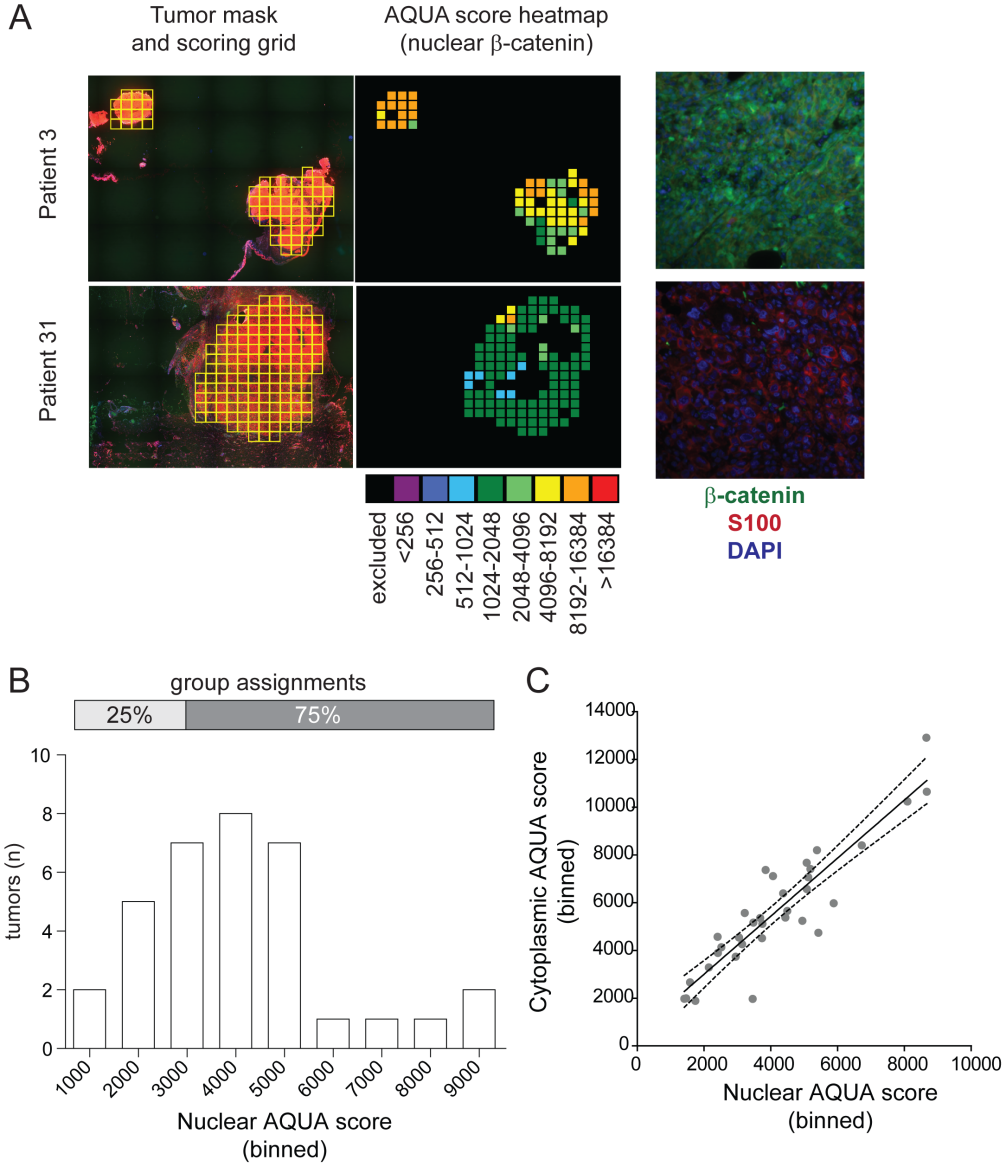
Patients on BRAFi exhibit a wide range of nuclear β-catenin expression. (A) Representative tumors with high (patient 3) and low (patient 31) levels of nuclear β-catenin are shown to illustrate the method of automated quantification (AQUA) used to stratify patients. On the left, a tumor mask grid is established based on staining of paraffin sections with an antibody targeting S100 (far left column). Nuclear β-catenin is then quantified by co-localization of β-catenin staining with the nuclear stain DAPI (right column), generating an AQUA score for each grid (second column) that is averaged to generate a mean AQUA score for each tumor. (B) This histogram depicts the distribution of mean nuclear β-catenin AQUA scores across the 32-patient BRAFi-treated cohort described in Table 1. Above, horizontal bars show the grouping exhibiting statistical significance on univariate analysis. (C) Mean AQUA scores for nuclear β-catenin were graphed against each tumor's AQUA score for cytoplasmic β-catenin (determined by automated quantification of β-catenin staining co-localized with S100 staining). Each tumor is represented by a gray dot, and the linear regression is shown (solid line) with 95% confidence intervals (dashed lines). The correlation coefficient (*r*) of 0.83 for tumors in this cohort indicates that mean AQUA scores for nuclear β-catenin correlate well with scores for cytoplasmic β-catenin, consistent with the model in which levels of both cytoplasmic and nuclear β-catenin can act as histological surrogates of Wnt/β-catenin activation.

Patients with metastatic melanoma carrying a *BRAF* mutation at the V600 position (confirmed by DNA sequencing) who received treatment with BRAFi (n = 32) were included in this study. Cohort characteristics are summarized in Table 1. The response rate was 72%, median time to progression was 16.3 weeks (95% CI: 13.9–18.6) and the median OS was 41.4 weeks (95% CI: 26.8–56.0). Automated quantification of immunohistochemical staining was used to measure mean nuclear β-catenin (Figure 1A-B). Mean scores for nuclear β-catenin ranged from 1411.4 to 8668.4 (Figure 1B). The ranked scores were stratified as shown in Figure 1B. Results using summed cytosolic and nuclear β-catenin were the same as results with nuclear β-catenin alone (data not shown), consistent with our observation that nuclear β-catenin scores correlate highly with cytoplasmic β-catenin scores within tumors (*r* = 0.83; Figure 1C).

**Table 1:** Characteristics of BRAFi patient cohort (n = 32).

Factor	Value	N	%
Total Patients	N	32	100%
Patient Sex	Female	12	38%
	Male	20	63%
Age at Trial Start (years)	Mean/Median (range)	52/57 (23–73)	-
Genotype	V600E	30	94%
	V600K	2	6%
BRAFi	Dabrafenib	29	91%
	Vemurafenib	3	9%
Active Brain Metastases at Trial Start	No	15	44%
	Yes	17	56%
Subsequent COMBI*	No	31	97%
	Yes	1	3%
M-stage	M1a	1	3%
	M1b	1	3%
	M1c	30	94%
Baseline Sum of Diameters (mm)	Mean/Median (range)	127/108(9–317)	-
ECOG	0	15	47%
	1	17	53%
LDH	Normal	15	47%
	Elevated	17	53%
Best CT Response	PD	1	3%
	SD	8	25%
	PR	23	72%
	CR	0	0%
Progression Status	Progressed	29	94%
	Not progressed	3	6%
BRAFi Status	Continuing BRAFi	28	88%
	Not on BRAFi	4	13%
Treatment Beyond Progression∧	No	17	53%
	Yes	15	47%
Treatment Beyond Progression (days)	Mean/Median (range)	116/66 (32–382)	-
Last Follow-up Status	Dead	22	72%
	Alive	10	28%
Follow-up (weeks)*	Mean/Median (range)	51/41 (8–153)	-

Abbreviations: PD,progressive disease; SD, stable disease; PR, partial response; CR, complete response.

*COMBI denotes subsequent enrolment in a clinical trial comparing combination BRAFi plus MEK inhibitor to placebo. Follow-up for subsequent COMBI patients (n = 1) was censored at date of cessation of mono-BRAFi.

∧Treatment beyond progression classified as cessation of BRAFi greater than 30 days after date of progression.

### Nuclear β-catenin and survival endpoints in patients treated with BRAFi

**Figure 2:**
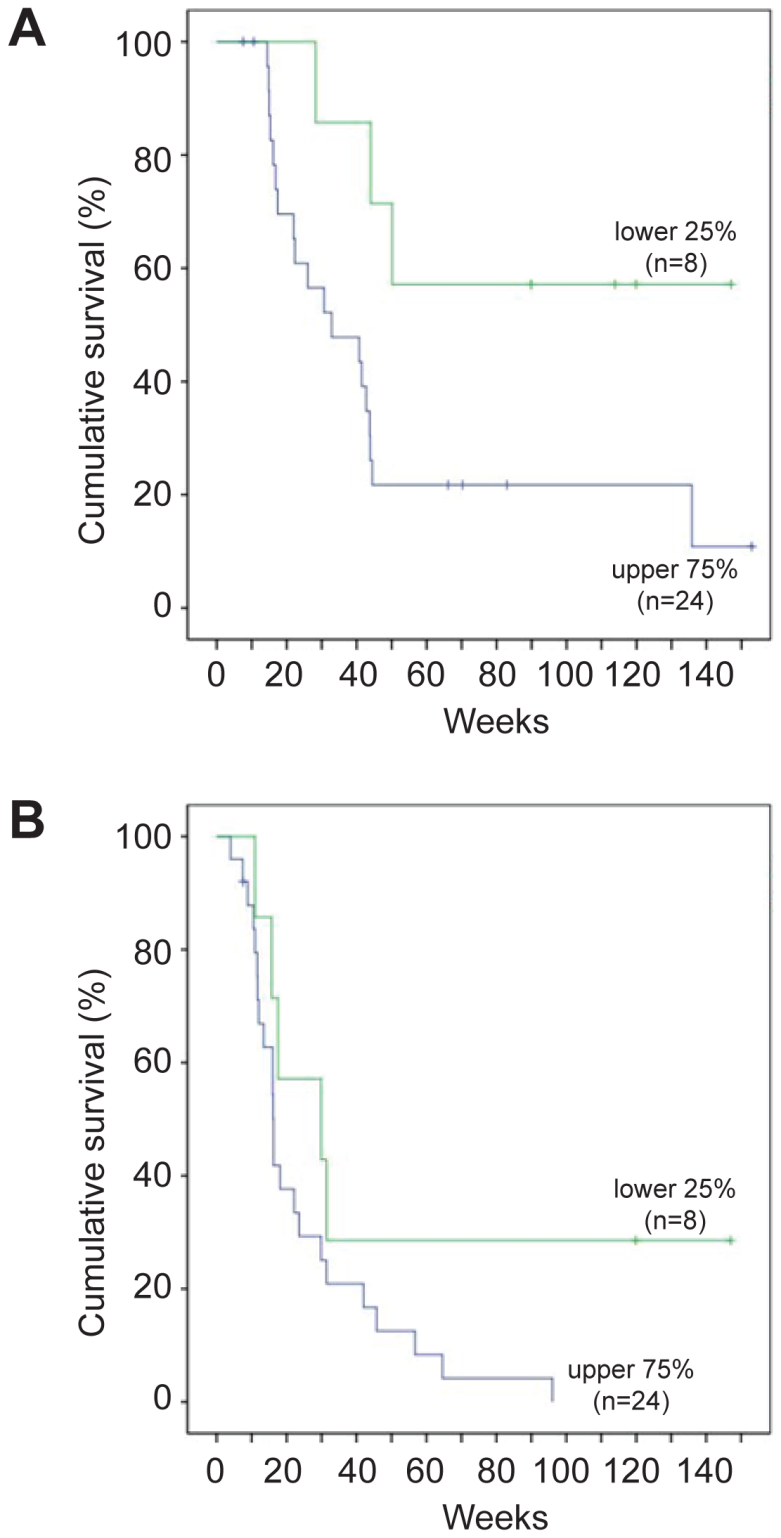
Lower levels of nuclear β-catenin significantly correlate with improved survival on BRAFi in post-hoc analysis. (A) In exploratory post-hoc analysis, patients with nuclear β-catenin scores in the lowest quartile exhibited a statistically-significant improvement in overall survival (p = 0.037) compared to the remainder of the cohort. (B) A similar trend towards improvement with patients in the lowest quartile was seen with progression-free survival, but did not reach statistical significance (p = 0.099).

Given the limited sample size, we performed post-hoc exploratory analysis comparing levels of nuclear β-catenin with the survival endpoints using different methods of patient stratification that subdivided the cohort into two or three groups. Comparison of patients in the lowest quartile of nuclear β-catenin (lowest 25%) to the remaining 75% of patients within the cohort revealed a significant difference in OS (p = 0.037; Figure 2A). A similar trend was observed with PFS using this stratification, although this was not statistically significant (Figure 2B (p = 0.099)). There was no significant difference between the groups for time to best RECIST CT response (data not shown).

### Long-term BRAFi treatment leads to down-regulation of cellular responses to WNT3A

**Figure 3:**
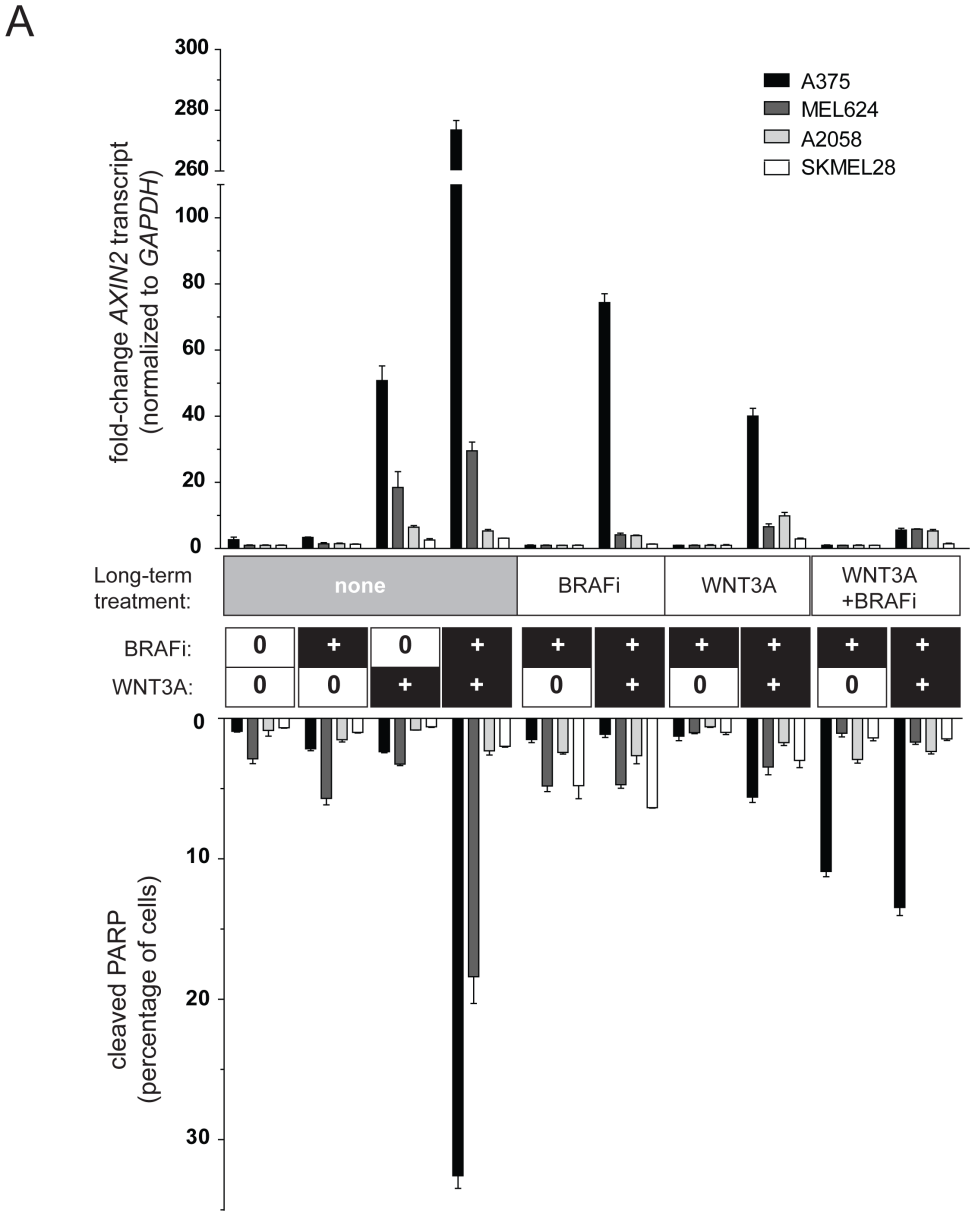
Long-term treatment with BRAFi downregulates cellular responses to WNT3A. Melanoma cell lines were cultured long-term (4–6 weeks) in 2 µM vemurafenib (BRAFi) in the absence or presence of continually-replenished WNT3A conditioned media (WNT3A and WNT3A + BRAFi). Cells were then treated with acutely with BRAF in the absence and presence of WNT3A conditioned media, and responses were compared to BRAFi-naïve cells (column sets 1–4). Transcriptional activation of Wnt/β-catenin signaling was assessed by quantitative-PCR-based measurements of the target gene *AXIN2*, normalized to *GAPDH* (upper bar sets). Cellular apoptosis was assessed through the detection of cleaved PARP by flow-cytometry (lower bar sets). Note that long-term treatment with BRAFi, WNT3A or WNT3A + BRAFi significantly attenuates activation of *AXIN2* transcription and enhancement of apoptosis by WNT3A. For each cell line, one-way ANOVA with Bonferroni's post-test was performed to determine statistical significance (see Table S1).

We treated a panel of melanoma cell lines for 4–6 weeks in the continued presence of 2 µM vemurafenib (BRAFi) and in the presence or absence of WNT3A (Figure 3). Two of these cell lines (A375 and MEL624) were previously shown to exhibit enhanced apoptosis in the presence of both WNT3A and BRAFi, while two of these cell lines (A2058 and SKMEL28) do not exhibit significant apoptosis with WNT3A and BRAFi [27]. These cells were compared to naïve cells that had not been chronically treated with BRAFi and/or WNT3A, but only acutely exposed to BRAFi and WNT3A overnight. Long-term treatment with BRAFi alone or BRAFi and/or WNT3A markedly inhibited activation of Wnt/β-catenin signaling as measured by *AXIN2* transcript levels (Figure 3, upper half). In parallel, we also measured the effects of acute exposure to BRAFi and/or WNT3A on apoptosis measured by cleaved PARP. Similarly, we saw that apoptosis with WNT3A and BRAFi was inhibited in cells chronically treated with BRAFi and/or WNT3A compared to naïve cells (Figure 3, lower half).

**Figure 4:**
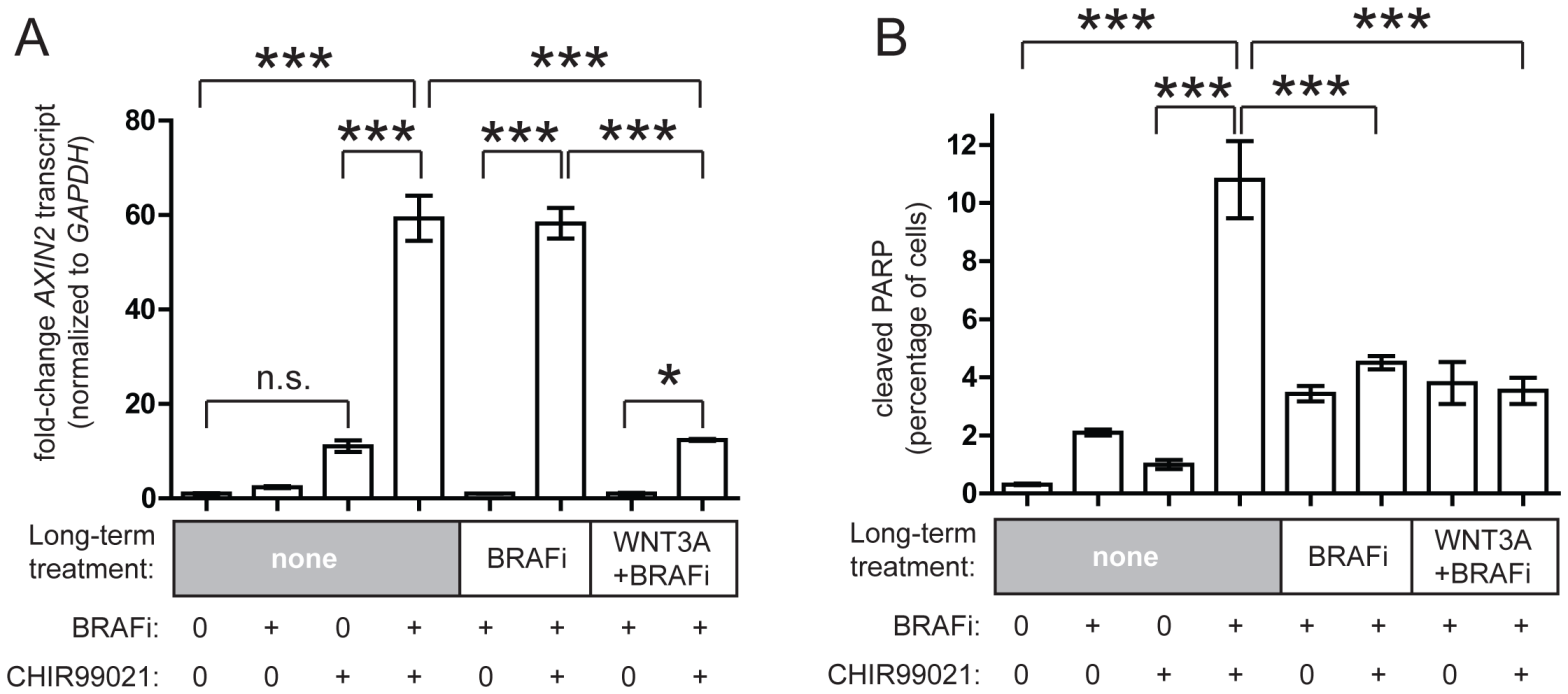
Long-term BRAFi treatment uncouples Wnt-mediated transcription from Wnt-mediated enhancement of apoptosis. (A) A375 cells treated long-term with BRAFi or WNT3A + BRAFi were then incubated overnight with CHIR99021. Long-term BRAFi treatment does not affect the ability of CHIR99021 to activate *AXIN2* transcription, but long-term WNT3A + BRAFi significantly decreases the upregulation of *AXIN2* transcription by CHIR99021. (B) In cells treated with long-term BRAFi, CHIR99021 is unable to further stimulate apoptosis with BRAFi. For these experiments, bars represent the mean and standard deviation of three distinct replicates for each condition. Data were compared using one-way ANOVA with Tukey's multiple comparison post-test, and relevant comparisons are shown (***, p<0.001; *, p<0.05; n.s., not significant). Data shown are representative of two to four experiments (each with three distinct replicate plates per condition) showing the same results.

Interestingly, long-term treatment of cells with BRAFi alone did not affect activation of Wnt/β-catenin signaling by the GSK3 inhibitor CHIR99021 (Figure 4A, compare columns 4 and 6), suggesting that the pathway is intact at the level of GSK3B, one of the key intracellular regulators of β-catenin abundance. However, cells cultured with BRAFi and WNT3A exhibited a highly blunted activation of Wnt/β-catenin signaling with CHIR99021 (Figure 4A, compare column 8 with columns 4 and 6). Unexpectedly, while long-term BRAFi alone did not inhibit activation of Wnt/β-catenin signaling by CHIR99021, it completely prevented any enhancement of apoptosis with CHIR99021 (Figure 4A and 4B, compare lane 4 to lane 6), similar to what was seen with WNT3A (Figure 3). A similar loss of CHIR99021-enhanced apoptosis was also seen in A375 cells treated chronically with BRAFi plus WNT3A (Figure 4B, lane 8). These results suggest that in melanoma cells exposed to long-term BRAFi, the transcriptional effects of Wnt/β-catenin signaling (measured by *AXIN2* transcript) are uncoupled from the enhancement of apoptosis by Wnt/β-catenin signaling at or above the level of GSK3B.

### Inhibition of ERK1/2 synergizes with WNT3A to promote melanoma cell apoptosis

**Figure 5:**
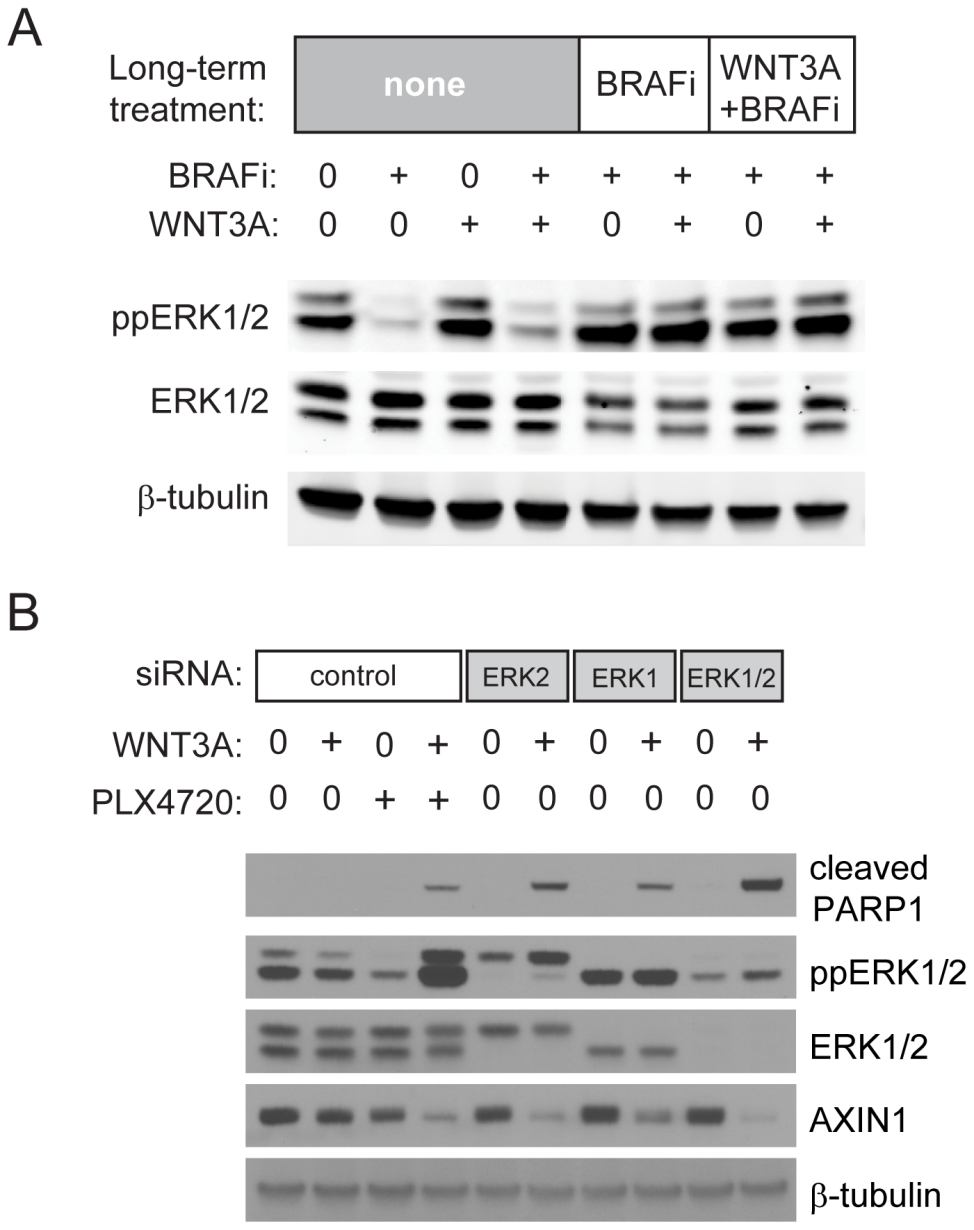
Activated ERK1 and ERK2 inhibit Wnt/β-catenin signaling in melanoma cells. (A) Levels of ERK1/2 phosphorylation (ppERK1/2) were compared by immunoblot in BRAFi-naïve A375 melanoma cells and A375 cells treated long-term for 4–6 weeks with BRAFi (2 µM vemurafenib) or WNT3A + BRAFi. Long-term treatment with BRAFi results in re-activation of ERK1/2 phosphorylation. No significant changes were seen in levels of total ERK1/2 or β-tubulin. (B) Human A375 melanoma cells were transfected with control siRNA or siRNA targeting ERK1 and ERK2, either individually or in combination (ERK1/2) at a concentration of 20nM. After 48 hours, transfected cells were then cultured overnight in the absence or presence of WNT3A conditioned media. For comparison, cells transfected with control siRNA were also treated with BRAFi (1 µM PLX4720). Apoptosis was measured by the cleavage of PARP1. Specific knockdown of ERK1 and ERK2 were visualized by loss of the appropriate bands detected using antibodies targeting phosphorylated ERK1/2 (ppERK1/2) or total ERK1/2. AXIN1 abundance decreased with knockdown of ERK1 and ERK2, either individually or in combination, paralleling the observed decrease in abundance seen with PLX4720.

As expected, long-term treatment of cells with BRAFi or BRAFi plus WNT3A is accompanied by reactivation of ERK1/2 phosphorylation (Figure 5A). Since reactivation of ERK1/2 represents a key downstream event during the acquisition of resistance to BRAFi, we explored whether ERK1/2 could regulate Wnt/β-catenin signaling in melanoma cells. We used siRNAs to selectively knock down levels of ERK1 and ERK2 (both individually and in combination) in human A375 melanoma cells homozygous for the *BRAF^V600E^* mutation (Figure 5B). In the absence of exogenously-added WNT3A, knockdown of either ERK1 or ERK2 individually did not result in significant apoptosis as measured on immunoblot by cleaved PARP1. Combining ERK1 and ERK2 siRNA led to PARP1 cleavage that was detectable with extended exposure (Figure 5B). In the presence of WNT3A, knockdown of ERK1 and ERK2 individually and in combination synergistically enhanced apoptosis to a degree that is similar to that observed with the BRAFi PLX4720 [28]. Additionally, knockdown of ERK1 and ERK2 in the presence of WNT3A markedly decreased abundance of the critical intracellular Wnt/β-catenin antagonist AXIN1 (Figure 5B), paralleling observations seen with pharmacological inhibition of BRAF and MEK and validating our prior siRNA-based identification of *MAPK3* and *MAPK1* as candidate regulators of Wnt/β-catenin signaling in melanoma cells [27], [29].

### Forced expression of BRAF^V600E^ enhances Wnt/β-catenin signaling in melanocytes

**Figure 6:**
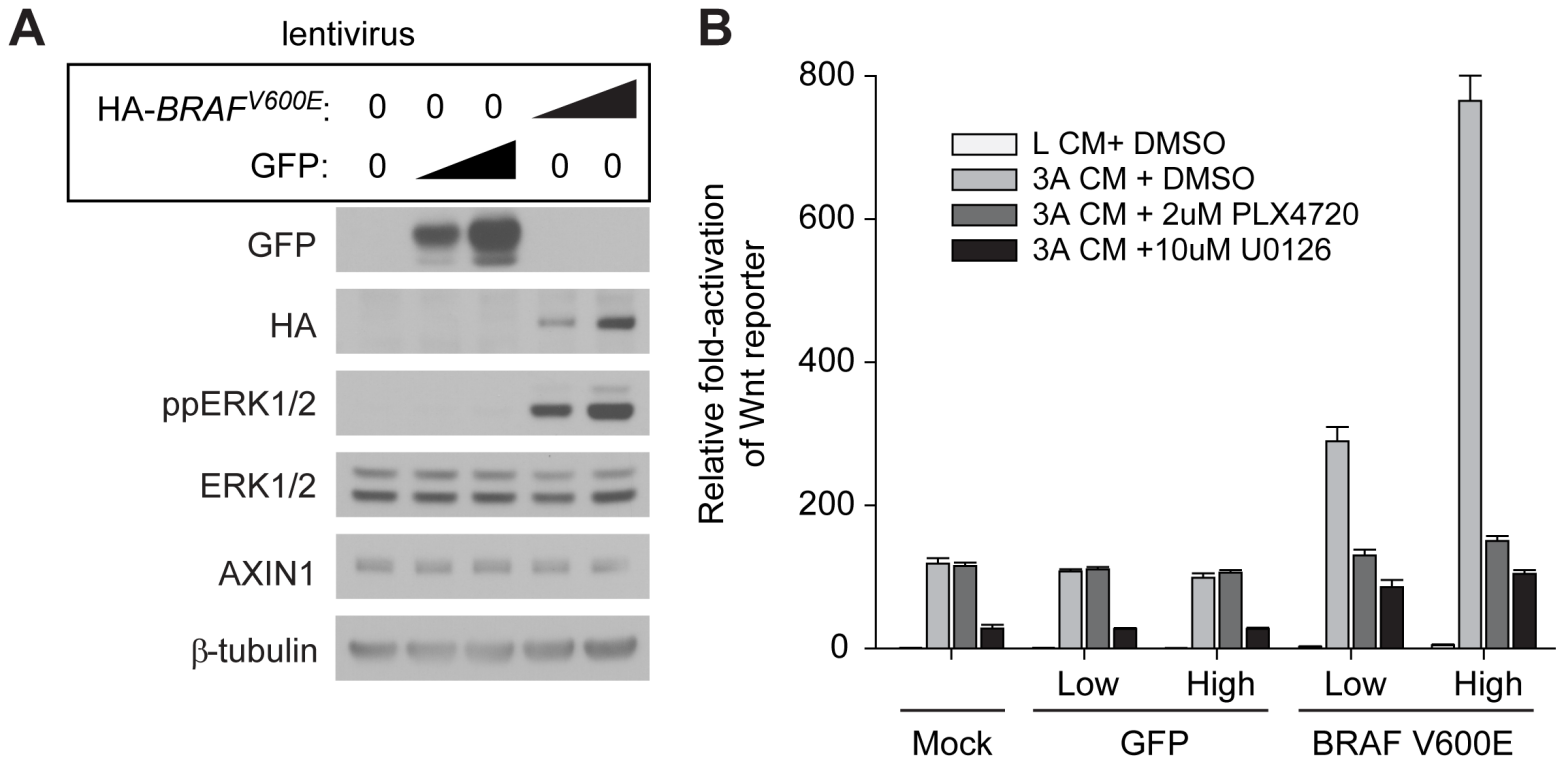
BRAF^V600E^ positively regulates Wnt/β-catenin signaling in melanocytes. A. Normal human melanocytes were transduced with lentivirus encoding either hemagglutinin (HA)-tagged BRAF^V600E^ (HA-BRAF^V600E^) or green fluorescent protein (GFP) at a low- and high-titer of virus. Expression of HA-BRAF^V600E^ was confirmed using an anti-HA antibody, while expression of GFP was confirmed using an anti-GFP antibody. Dose-dependent activation of ERK1/2 phosphorylation (ppERK1/2) confirmed activation of MAPK signaling with HA-BRAF^V600E^, but not GFP. No significant change in AXIN1 abundance was seen. B. Normal human melanocytes stably expressing a transduced β-catenin-activated reporter (BAR; see [40]) were transduced with lentivirus encoding either GFP or HA-BRAF^V600E^, and then treated with control L-cell conditioned media (LCM) or WNT3A-conditioned media (3A CM) combined with either DMSO vehicle, the BRAFi PLX4720, or the MEK inhibitor U0126. Expression of HA-BRAF^V600E^ enhanced activation of Wnt/β-catenin signaling in a dose-dependent manner (arrows), and this activation was completely inhibited by PLX4720 and U0126. In the absence of transduced HA-BRAF^V600E^, U0126 treatment inhibited activation of the reporter by 3A CM, likely reflecting the inhibition of low baseline levels of MAPK signaling. Data are representative of three experiments with similar results.

Normal human melanocytes (which normally do not exhibit activating mutations in *BRAF*) were transduced with lentiviral constructs encoding either GFP or an epitope-tagged BRAF^V600E^ (Figure 6). Forced expression of *BRAF^V600E^* (but not GFP) led to a dose-dependent increase in phosphorylation of ERK1/2, confirming increased activation of the MAPK signaling cascade (Figure 6A). While activation of ERK1/2 by BRAF^V600E^ in melanoma cells *inhibits* Wnt/β-catenin signaling [27], forced expression of BRAF^V600E^ *enhances* Wnt/β-catenin signaling in normal melanocytes (Figure 6B). The activation of Wnt/β-catenin signaling in normal melanocytes by BRAF^V600E^ is inhibited by BRAFi (PLX4720) or inhibition of MEK using U0126 (Figure 6B). These results indicate that the cross-talk between Wnt/β-catenin and MAPK signaling in melanoma cells is distinct from the interaction between these two pathways in non-transformed melanocytes.

## Discussion

This study unexpectedly found that *increased* nuclear β-catenin in biopsies taken prior to commencing BRAFi therapy is associated with *decreased* survival in patients treated with BRAFi. Decreased Wnt/β-catenin signaling was seen in patient tumors after progression on BRAFi, which may result from negative regulation of Wnt/β-catenin signaling by ERK1/2 reactivation following the acquisition of BRAFi resistance. The inhibition of Wnt/β-catenin signaling by BRAF activation in melanoma cells was not observed in normal melanocytes, consistent with a model in which MAPK-mediated disruption of homeostatic Wnt/β-catenin signaling can contribute to melanoma progression.

While Wnt/β-catenin signaling has not been the focus of existing therapeutic efforts in melanoma, there is accumulating evidence that signaling cross-talk between this pathway and BRAF/MAPK signaling influences melanoma progression. We directly investigated how Wnt/β-catenin signaling in patient tumors could impact clinical response to BRAFi, and our unanticipated results suggest that prediction of BRAFi treatment response may not simply correlate to apoptosis as has been previously suggested on the basis of the results of experiments utilizing existing pre-clinical laboratory models. Studies using patient cohorts prior to the advent of both routine *BRAF* genotyping and BRAFi correlated *increased* nuclear or cytoplasmic β-catenin with *improved* survival [22]–[24], so this current result raises the question as to whether patients with *BRAF*-mutant melanomas exhibiting high levels of nuclear β-catenin could potentially do better with other therapies or combinations of targeted drugs.

As with any small biomarker study these results should be interpreted with caution, and a larger study utilizing an independent patient cohort is needed to address whether the trend in OS seen with post-hoc analysis is truly significant. This study was limited to melanoma patients with stage IV disease, half of whom had brain metastases at the time of diagnosis, so these findings may not necessarily reflect what would be seen in patients with earlier-stage disease. Comparison of this cohort to previously published survival studies measuring nuclear or cytoplasmic β-catenin in molecularly uncharacterized melanoma tumors remains limited and unavoidably indirect, since these earlier studies did not account for the status of *BRAF* or *NRAS*. Furthermore, the molecular characterization of the melanomas in this study is limited with regards to the mutational status of other genes implicated in melanoma biology. Finally, our study does not account for the mechanisms underlying the emergence of resistance in individual patients, which may differentially impact Wnt/β-catenin signaling.

**Figure 7:**
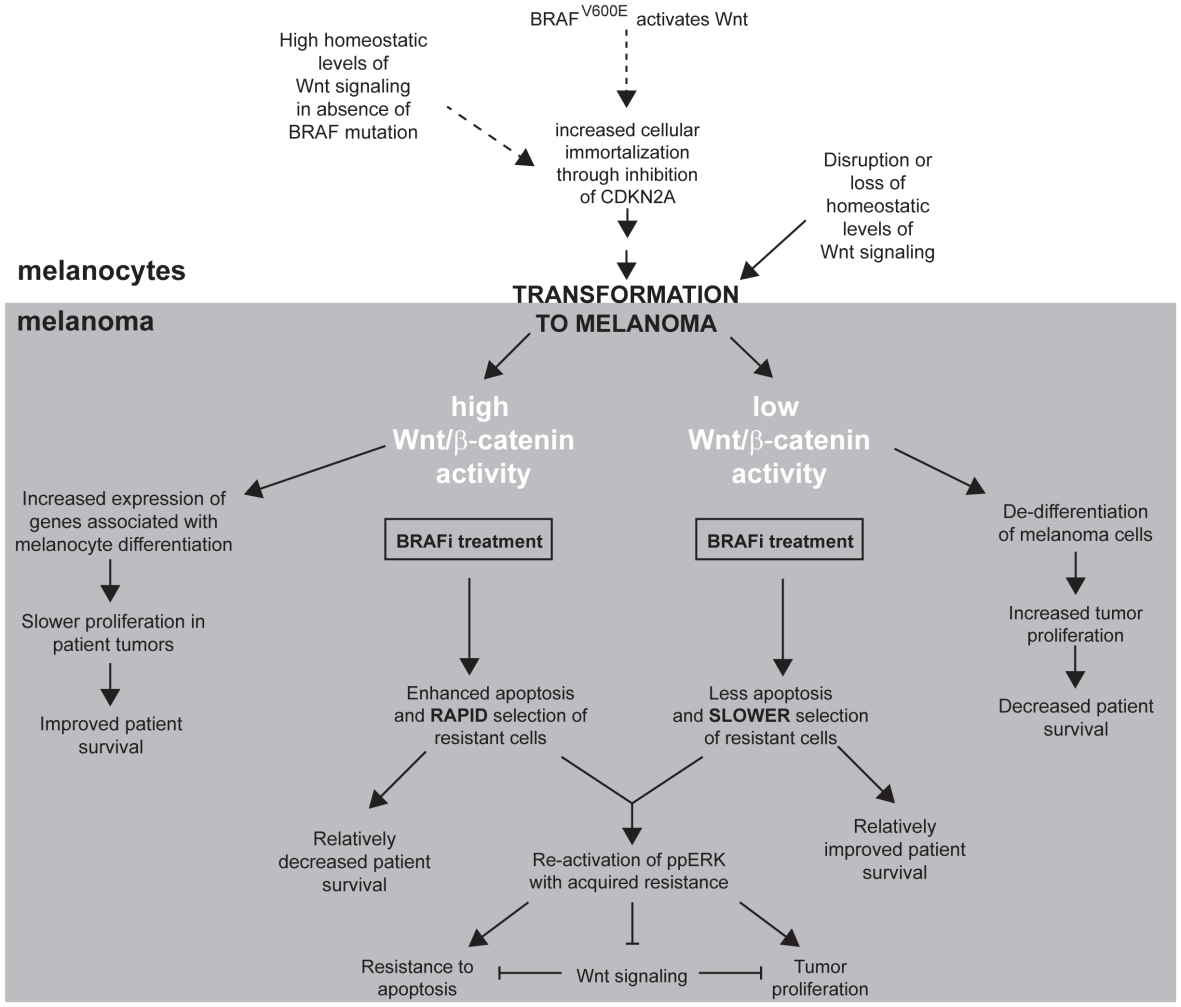
An evolving model for Wnt/β-catenin signaling in melanoma. This chart integrates results from existing transgenic mouse and human melanoma studies, as well as results from this study, into an evolving unified model that can be further tested for refinement. Dashed lines/arrows indicate that studies leading to this part of the model utilized overexpression of a mutant (non-degradable) β-catenin in transgenic mice, which may or may not be applicable in patient melanomas where these mutations are quite rare [31]. This model accounts for the clinical observation in multiple studies of improved survival with elevated nuclear β-catenin in tumors from molecularly unselected melanoma patients. In melanocytes as well as nevi, high levels of nuclear β-catenin and Wnt signaling are observed, and the loss of nuclear β-catenin correlates with progression from benign melanocytic lesions to melanoma.

Our observation that cross-talk between Wnt/β-catenin and MAPK signaling in melanoma cells is opposite of what occurs in non-transformed melanocytes suggests that molecular events during early melanomagenesis can significantly alter the mechanisms by which these pathways cooperate to regulate cellular function. Understanding the molecular mechanisms of how Wnt/β-catenin signaling is disrupted during the early stages of melanoma may potentially uncover novel avenues of therapeutic targeting that may be important not only for optimizing melanoma treatment, but also for developing strategies aimed at melanoma prevention and the pathologic distinction of nevi from melanomas which can be extremely challenging [30]. To date, it has been difficult to organize collective data from mouse models, human melanoma cell lines and patients into a consistent unified model [31]. It is possible that differences in how the cross-talk between these two pathways is regulated temporally throughout the process of melanomagenesis could differ between mouse and human models, particularly since studies in mouse models utilize forced expression of a non-degradable β-catenin mutant that is rarely found in patient tumors. Figure 7 provides a working model for how the interaction between Wnt/β-catenin and MAPK signaling could account for our current results in the context of previous observations.

The initial enhancement of apoptosis by the combination of high Wnt/β-catenin signaling plus BRAFi may more rapidly cultivate an aggressive cell population in patients, which would certainly be consistent with our current observations. While activation of Wnt/β-catenin signaling in combination with BRAFi enhances apoptosis in cultured cell models, the degree of cell death is not 100% [27]. This model predicts increased apoptosis in tumor cells with high levels of Wnt/β-catenin signaling upon BRAFi treatment, while cells with decreased Wnt/β-catenin signaling would be more resistant and therefore enriched following BRAFi. If this model is indeed true, it would suggest that effective combination therapies may need to demonstrate near-complete activation of cellular apoptosis in pre-clinical models to result in predictable improvements in patient survival outcomes. Whether activators of Wnt/β-catenin signaling could be part of these combinations in certain patient populations requires further study.

Recent studies in mouse models have suggested that the presence of active Wnt/β-catenin signaling may be permissive to metastasis in the context of BRAF/MAPK activation [32], [33]. It is possible that increased Wnt/β-catenin signaling upon inhibition of mutant BRAF^V600E^ may negatively impact patient survival by permitting or enhancing metastatic spread in certain cell populations within the tumor. Regulation of host immune responses may also play a role given recent observations that constitutive activation of Wnt/β-catenin signaling in melanoma cells can negatively regulate anti-tumor immune responses in a mouse model [34]. Again, larger studies utilizing patient samples derived following clinical responses to immunotherapy with BRAFi could further clarify the relevance of Wnt/β-catenin signaling and crosstalk with MAPK signaling in this context.

The results from this study highlight the difficulty with extrapolating results from laboratory models to patients treated with BRAFi, particularly with pathways like Wnt/β-catenin and MAPK signaling that exhibit context-dependent reciprocal regulation. Given the unexpected lack of correlation between β-catenin staining and patient outcome in this molecularly-selected study, future studies of both targeted BRAFi and targeted MEK inhibitors should consider quantifying levels of nuclear β-catenin to assess whether this biomarker may represent an important determinant for optimizing and individualizing the treatment of patients with metastatic melanoma. Larger studies with data from both pre- and post-treatment samples linked to mutation status and clinical response could also address whether the suppression of Wnt/β-catenin signaling by activated BRAF (and ERK1/2) contributes to the decreased survival observed in patients with tumors harboring *BRAF^V600E^* mutations. Future studies can also clarify whether the disease progression accompanied by re-activation of ERK1/2 during the development of resistance involves the suppression of Wnt/β-catenin signaling. The answers to these questions will help illuminate if, how and when the therapeutic manipulation of Wnt/β-catenin signaling could be potentially leveraged to enhance existing clinical strategies involving BRAFi.

## Materials and Methods

### Ethics Statement

Patient specimens were formalin-fixed, paraffin-embedded tumors. Informed written consent was obtained for each patient under approved protocols (Protocol No X10-0305 &HREC/10/RPAH/539 and Protocol No X10-0300 HREC/10/RPAH/530) governed by the Human Research Ethics Committee of the Royal Prince Albert Hospital (Sydney NSW, Australia). All clinical investigation was conducted according to principles outlined in the Declaration of Helsinki.

### Cell lines and biochemical reagents

Normal human melanocytes were obtained commercially from Life Technologies (Grand Island, NY) and cultured using the vendor's suggested media and culture conditions. Melanoma cell lines and their culture conditions have been previously described [27]. All cell lines were cultured in the presence of 5 µg/ml of Plasmocin from Invivogen (San Diego, CA), and negative Mycoplasma status was accomplished through interval surveillance with the Mycofluor assay kit from Life Technologies (Grand Island, NY) as previously published [23]. WNT3A-conditioned media was generated as previously described [27]. The plasmid encoding hemagglutinin (HA)-tagged BRAF^V600E^ was purchased from Biomyx (San Diego, CA). The coding sequence for HA-BRAF^V600E^ was inserted using standard cloning techniques into third-generation replication-deficient lentivirus (described in [23]). Viral particles were harvested from supernatants of transfected HEK293 cells and used at varying titers to infect human melanocytes over the course of two days. Apoptosis was measured by detection of cleaved PARP1 [35] on immunoblots using an antibody from Cell Signaling Technologies (Danvers, MA). Antibody-mediated detection of AXIN1, phospho-ERK1/2 and total ERK1/2 was performed as previously described [27]. The siRNA duplexes targeting ERK1 and ERK2 were obtained from Ambion/Life Technologies (Grand Island, NY). Transfections of siRNAs were performed using RNAiMax from Invitrogen/Life Technologies (Grand Island, NY). Proteins were separated by Nu-PAGE electrophoresis on commercially-prepared gradient gels from Life Technologies (Grand Island, NY), and subsequently immobilized by transfer to nitrocellulose membranes. Visualization of all immunoblots was performed using film-based detection of enhanced chemiluminescence from Pierce (Rockford, IL). Immunoblots presented in this manuscript are representative of three or more distinct experiments.

### Patients

Patients were selected on the basis of availability of baseline melanoma tumor samples. All patients received a BRAFi via enrolment in a clinical trial; either the GlaxoSmithKline (GSK) Phase 1/2 trial of dabrafenib (12 patients) [12], the GSK phase 2 trial of dabrafenib (3 patients) [36], the GSK phase 2 trial of patients with active brain metastases (14 patients) [37] or the Roche Phase 2 or 3 trial of vemurafenib (3 patients) [11], [14]. All patients treated with vemurafenib received 960mg twice daily. All patients treated with dabrafenib received ≥ the daily recommended phase 2 dose of 300mg after first computed axial tomography (CT) scan.

### Response to Treatment and Clinical Outcome

Objective response to BRAFi treatment was assessed with CT scanning 6–9 weekly, using RECIST 1.0 [38] for those on the phase 1/2 study of dabrafenib, and RECIST 1.1 [39] for all other patients. Three survival outcomes were tested using the Kaplan-Meier method together with the Log Rank test; time to best response, progression-free survival, and overall survival (Figures 2). All time intervals were measured in relation to the commencement of BRAFi. The primary endpoints for this study were overall survival (OS), progression-free survival (PFS) and time to best response. For secondary endpoints, best computerized tomography (CT) response was assessed categorically as progressive or stable disease versus partial response (no patients had a complete response), and also as best percent-change in RECIST target lesions. Follow-up for one patient taking subsequent COMBI therapy was censored at date of cessation of BRAFi. The Mann-Whitney U test was used to address correlations between nuclear β-catenin and RECIST criteria.

### Histological quantification of β-catenin in patient tumors

Immunohistochemical cytoplasmic and nuclear β-catenin stains were conducted on tumor samples (either primary melanomas or melanoma metastases) from this cohort collected prior to the initiation of BRAFi. Five micron-thick tumor sections were labeled immunohistochemically with a mouse monoclonal antibody to β-catenin (BD Transduction Laboratories, San Jose CA; catalog number 610154) at a dilution of 1∶1000, which was experimentally determined to be the optimal concentration using standardized specimens. Fluorescent visualization was performed using an anti-mouse Cy5-conjugated antibody (DAKO, Carpinteria CA). Nuclei were visualized using Prolong Gold DAPI (Life Sciences, Grand Island NY). Initial tumor specimens were examined, dissected, processed and interpreted at the Melanoma Institute of Australia prior to antigen retrieval, immunostaining, quantification and imaging of these samples by HistoRX (Branford CT), who were blinded to all outcomes data. S100 staining was used to identify a tumor mask, defined as the cellular area of the tumor (Figure 1), and the subsequent AQUA scoring grids were established by certified pathologists. Subsequent AQUA scores were obtained for each grid and averaged for each tumor, with nuclear and cytoplasmic compartments defined by DAPI and S100, respectively.

### Statistical analysis

Scores for cytoplasmic and nuclear β-catenin for each patient were ranked and stratified into five groups *a priori* by the team that conducted the stains, blinded to the clinical outcome data. Scores for nuclear β-catenin were averaged for each tumor based on an average signal from each tumor grid (Figure 1). The GraphPad Prism version 5.0 software suite (GraphPad Software, La Jolla CA) and the IBM SPSS v21 software package (SPSS, Chicago IL) were utilized for statistical analysis. All p-values less than 0.05 were considered statistically significant. Univariate survival analyses were conducted with the Kaplan-Meier method together with the Log Rank test. Bivariate correlations were run using the Mann Whitney U test or Spearman's correlation where appropriate. All time intervals were measured in relation to the commencement of BRAFi. The primary endpoints for this study were overall survival (OS), progression-free survival (PFS) and time to best response. For secondary endpoints, best computerized tomography (CT) response was assessed categorically as progressive or stable disease versus partial response (no patients had a complete response), and also as best percent-change in RECIST target lesions. Overall survival for five patients having subsequent targeted/immune therapy was censored at the time of cessation of BRAFi.

## Supporting Information

Table S1 **These tables show the results of one-way ANOVA for** **Figure 3** **, with post-test p-values indicated for each cell line.**
(DOC)
