# Supplementary material for: Targeted BRAF Inhibition Impacts Survival in Melanoma Patients with High Levels of Wnt/β-Catenin Signaling
Source: PLoS One. 2014 Apr 14;9(4):e94748. doi: 10.1371/journal.pone.0094748 (PMC3986217; doi:10.1371/journal.pone.0094748)
Supplement: Table S1 — These tables show the results of one-way ANOVA for Figure 3 , with post-test p-values indicated for each cell line. (DOC) [file pone.0094748.s001.doc]

***Supplemental Table S1.***

One-way ANOVA and post-test results shown for all cell lines in Figure 3.

| Table Analyzed | isolated A375 AXIN2 |  |  |  |  |
| --- | --- | --- | --- | --- | --- |
|  |  |  |  |  |  |
| One-way analysis of variance |  |  |  |  |  |
| P value | < 0.0001 |  |  |  |  |
| P value summary | *** |  |  |  |  |
| Are means signif. different? (P < 0.05) | Yes |  |  |  |  |
| Number of groups | 10 |  |  |  |  |
| F | 1653 |  |  |  |  |
| R squared | 0.9987 |  |  |  |  |
|  |  |  |  |  |  |
| ANOVA Table | SS | df | MS |  |  |
| Treatment (between columns) | 192100 | 9 | 21340 |  |  |
| Residual (within columns) | 258.2 | 20 | 12.91 |  |  |
| Total | 192300 | 29 |  |  |  |
|  |  |  |  |  |  |
| Bonferroni's Multiple Comparison Test | Mean Diff. | t | Significant? P < 0.05? | Summary | 95% CI of diff |
| parental (L+DMSO) vs parental (L+BRAFi) | -0.6774 | 0.2309 | No | ns | -11.84 to 10.48 |
| parental (L+DMSO) vs parental (W3A) | -48.13 | 16.41 | Yes | *** | -59.29 to -36.97 |
| parental (L+DMSO) vs parental (W3A+BRAFi) | -270.8 | 92.32 | Yes | *** | -282.0 to -259.7 |
| parental (L+DMSO) vs BRAFi (L+BRAFi) | 1.667 | 0.5682 | No | ns | -9.494 to 12.83 |
| parental (L+DMSO) vs BRAFi (W3A+BRAFi) | -71.65 | 24.42 | Yes | *** | -82.81 to -60.49 |
| parental (L+DMSO) vs W3A (L+BRAFi) | 1.667 | 0.5682 | No | ns | -9.494 to 12.83 |
| parental (L+DMSO) vs W3A (W3A+BRAFi) | -37.36 | 12.74 | Yes | *** | -48.52 to -26.20 |
| parental (L+DMSO) vs W3A+BRAFi (L+BRAFi) | 1.667 | 0.5682 | No | ns | -9.494 to 12.83 |
| parental (L+DMSO) vs W3A+BRAFi (W3A+BRAFi) | -2.927 | 0.9976 | No | ns | -14.09 to 8.234 |
| parental (L+BRAFi) vs parental (W3A) | -47.45 | 16.17 | Yes | *** | -58.61 to -36.29 |
| parental (L+BRAFi) vs parental (W3A+BRAFi) | -270.2 | 92.09 | Yes | *** | -281.3 to -259.0 |
| parental (L+BRAFi) vs BRAFi (L+BRAFi) | 2.344 | 0.7991 | No | ns | -8.816 to 13.50 |
| parental (L+BRAFi) vs BRAFi (W3A+BRAFi) | -70.97 | 24.19 | Yes | *** | -82.13 to -59.81 |
| parental (L+BRAFi) vs W3A (L+BRAFi) | 2.344 | 0.7991 | No | ns | -8.816 to 13.50 |
| parental (L+BRAFi) vs W3A (W3A+BRAFi) | -36.68 | 12.50 | Yes | *** | -47.85 to -25.52 |
| parental (L+BRAFi) vs W3A+BRAFi (L+BRAFi) | 2.344 | 0.7991 | No | ns | -8.816 to 13.50 |
| parental (L+BRAFi) vs W3A+BRAFi (W3A+BRAFi) | -2.249 | 0.7667 | No | ns | -13.41 to 8.911 |
| parental (W3A) vs parental (W3A+BRAFi) | -222.7 | 75.92 | Yes | *** | -233.9 to -211.6 |
| parental (W3A) vs BRAFi (L+BRAFi) | 49.79 | 16.97 | Yes | *** | 38.63 to 60.96 |
| parental (W3A) vs BRAFi (W3A+BRAFi) | -23.52 | 8.018 | Yes | *** | -34.68 to -12.36 |
| parental (W3A) vs W3A (L+BRAFi) | 49.79 | 16.97 | Yes | *** | 38.63 to 60.96 |
| parental (W3A) vs W3A (W3A+BRAFi) | 10.77 | 3.670 | No | ns | -0.3954 to 21.93 |
| parental (W3A) vs W3A+BRAFi (L+BRAFi) | 49.79 | 16.97 | Yes | *** | 38.63 to 60.96 |
| parental (W3A) vs W3A+BRAFi (W3A+BRAFi) | 45.20 | 15.41 | Yes | *** | 34.04 to 56.36 |
| parental (W3A+BRAFi) vs BRAFi (L+BRAFi) | 272.5 | 92.89 | Yes | *** | 261.3 to 283.7 |
| parental (W3A+BRAFi) vs BRAFi (W3A+BRAFi) | 199.2 | 67.90 | Yes | *** | 188.0 to 210.4 |
| parental (W3A+BRAFi) vs W3A (L+BRAFi) | 272.5 | 92.89 | Yes | *** | 261.3 to 283.7 |
| parental (W3A+BRAFi) vs W3A (W3A+BRAFi) | 233.5 | 79.59 | Yes | *** | 222.3 to 244.6 |
| parental (W3A+BRAFi) vs W3A+BRAFi (L+BRAFi) | 272.5 | 92.89 | Yes | *** | 261.3 to 283.7 |
| parental (W3A+BRAFi) vs W3A+BRAFi (W3A+BRAFi) | 267.9 | 91.32 | Yes | *** | 256.8 to 279.1 |
| BRAFi (L+BRAFi) vs BRAFi (W3A+BRAFi) | -73.32 | 24.99 | Yes | *** | -84.48 to -62.15 |
| BRAFi (L+BRAFi) vs W3A (L+BRAFi) | -0.0000001192 | 0.00000004064 | No | ns | -11.16 to 11.16 |
| BRAFi (L+BRAFi) vs W3A (W3A+BRAFi) | -39.03 | 13.30 | Yes | *** | -50.19 to -27.87 |
| BRAFi (L+BRAFi) vs W3A+BRAFi (L+BRAFi) | -0.0000002384 | 0.00000008127 | No | ns | -11.16 to 11.16 |
| BRAFi (L+BRAFi) vs W3A+BRAFi (W3A+BRAFi) | -4.593 | 1.566 | No | ns | -15.75 to 6.567 |
| BRAFi (W3A+BRAFi) vs W3A (L+BRAFi) | 73.32 | 24.99 | Yes | *** | 62.15 to 84.48 |
| BRAFi (W3A+BRAFi) vs W3A (W3A+BRAFi) | 34.29 | 11.69 | Yes | *** | 23.13 to 45.45 |
| BRAFi (W3A+BRAFi) vs W3A+BRAFi (L+BRAFi) | 73.32 | 24.99 | Yes | *** | 62.15 to 84.48 |
| BRAFi (W3A+BRAFi) vs W3A+BRAFi (W3A+BRAFi) | 68.72 | 23.43 | Yes | *** | 57.56 to 79.88 |
| W3A (L+BRAFi) vs W3A (W3A+BRAFi) | -39.03 | 13.30 | Yes | *** | -50.19 to -27.87 |
| W3A (L+BRAFi) vs W3A+BRAFi (L+BRAFi) | -0.0000001192 | 0.00000004064 | No | ns | -11.16 to 11.16 |
| W3A (L+BRAFi) vs W3A+BRAFi (W3A+BRAFi) | -4.593 | 1.566 | No | ns | -15.75 to 6.567 |
| W3A (W3A+BRAFi) vs W3A+BRAFi (L+BRAFi) | 39.03 | 13.30 | Yes | *** | 27.87 to 50.19 |
| W3A (W3A+BRAFi) vs W3A+BRAFi (W3A+BRAFi) | 34.44 | 11.74 | Yes | *** | 23.27 to 45.60 |
| W3A+BRAFi (L+BRAFi) vs W3A+BRAFi (W3A+BRAFi) | -4.593 | 1.566 | No | ns | -15.75 to 6.567 |

| Table Analyzed | isolated MEL624 AXIN2 |  |  |  |  |
| --- | --- | --- | --- | --- | --- |
|  |  |  |  |  |  |
| One-way analysis of variance |  |  |  |  |  |
| P value | < 0.0001 |  |  |  |  |
| P value summary | *** |  |  |  |  |
| Are means signif. different? (P < 0.05) | Yes |  |  |  |  |
| Number of groups | 10 |  |  |  |  |
| F | 29.55 |  |  |  |  |
| R squared | 0.9301 |  |  |  |  |
|  |  |  |  |  |  |
| ANOVA Table | SS | df | MS |  |  |
| Treatment (between columns) | 2476 | 9 | 275.1 |  |  |
| Residual (within columns) | 186.2 | 20 | 9.308 |  |  |
| Total | 2662 | 29 |  |  |  |
|  |  |  |  |  |  |
| Bonferroni's Multiple Comparison Test | Mean Diff. | t | Significant? P < 0.05? | Summary | 95% CI of diff |
| parental (L+DMSO) vs parental (L+BRAFi) | -0.4440 | 0.1782 | No | ns | -9.921 to 9.033 |
| parental (L+DMSO) vs parental (W3A) | -17.45 | 7.005 | Yes | *** | -26.93 to -7.973 |
| parental (L+DMSO) vs parental (W3A+BRAFi) | -28.58 | 11.47 | Yes | *** | -38.05 to -19.10 |
| parental (L+DMSO) vs BRAFi (L+BRAFi) | -0.0000001192 | 0.00000004786 | No | ns | -9.477 to 9.477 |
| parental (L+DMSO) vs BRAFi (W3A+BRAFi) | -3.142 | 1.261 | No | ns | -12.62 to 6.335 |
| parental (L+DMSO) vs W3A (L+BRAFi) | 0.00000005961 | 0.00000002393 | No | ns | -9.477 to 9.477 |
| parental (L+DMSO) vs W3A (W3A+BRAFi) | -5.585 | 2.242 | No | ns | -15.06 to 3.892 |
| parental (L+DMSO) vs W3A+BRAFi (L+BRAFi) | -0.0000002384 | 0.00000009571 | No | ns | -9.477 to 9.477 |
| parental (L+DMSO) vs W3A+BRAFi (W3A+BRAFi) | -4.865 | 1.953 | No | ns | -14.34 to 4.612 |
| parental (L+BRAFi) vs parental (W3A) | -17.01 | 6.827 | Yes | *** | -26.48 to -7.529 |
| parental (L+BRAFi) vs parental (W3A+BRAFi) | -28.13 | 11.29 | Yes | *** | -37.61 to -18.65 |
| parental (L+BRAFi) vs BRAFi (L+BRAFi) | 0.4440 | 0.1782 | No | ns | -9.033 to 9.921 |
| parental (L+BRAFi) vs BRAFi (W3A+BRAFi) | -2.698 | 1.083 | No | ns | -12.17 to 6.779 |
| parental (L+BRAFi) vs W3A (L+BRAFi) | 0.4440 | 0.1782 | No | ns | -9.033 to 9.921 |
| parental (L+BRAFi) vs W3A (W3A+BRAFi) | -5.141 | 2.064 | No | ns | -14.62 to 4.336 |
| parental (L+BRAFi) vs W3A+BRAFi (L+BRAFi) | 0.4440 | 0.1782 | No | ns | -9.033 to 9.921 |
| parental (L+BRAFi) vs W3A+BRAFi (W3A+BRAFi) | -4.421 | 1.775 | No | ns | -13.90 to 5.056 |
| parental (W3A) vs parental (W3A+BRAFi) | -11.13 | 4.466 | Yes | * | -20.60 to -1.649 |
| parental (W3A) vs BRAFi (L+BRAFi) | 17.45 | 7.005 | Yes | *** | 7.973 to 26.93 |
| parental (W3A) vs BRAFi (W3A+BRAFi) | 14.31 | 5.744 | Yes | *** | 4.831 to 23.78 |
| parental (W3A) vs W3A (L+BRAFi) | 17.45 | 7.005 | Yes | *** | 7.973 to 26.93 |
| parental (W3A) vs W3A (W3A+BRAFi) | 11.87 | 4.763 | Yes | ** | 2.388 to 21.34 |
| parental (W3A) vs W3A+BRAFi (L+BRAFi) | 17.45 | 7.005 | Yes | *** | 7.973 to 26.93 |
| parental (W3A) vs W3A+BRAFi (W3A+BRAFi) | 12.58 | 5.052 | Yes | ** | 3.108 to 22.06 |
| parental (W3A+BRAFi) vs BRAFi (L+BRAFi) | 28.58 | 11.47 | Yes | *** | 19.10 to 38.05 |
| parental (W3A+BRAFi) vs BRAFi (W3A+BRAFi) | 25.43 | 10.21 | Yes | *** | 15.96 to 34.91 |
| parental (W3A+BRAFi) vs W3A (L+BRAFi) | 28.58 | 11.47 | Yes | *** | 19.10 to 38.05 |
| parental (W3A+BRAFi) vs W3A (W3A+BRAFi) | 22.99 | 9.229 | Yes | *** | 13.51 to 32.47 |
| parental (W3A+BRAFi) vs W3A+BRAFi (L+BRAFi) | 28.58 | 11.47 | Yes | *** | 19.10 to 38.05 |
| parental (W3A+BRAFi) vs W3A+BRAFi (W3A+BRAFi) | 23.71 | 9.518 | Yes | *** | 14.23 to 33.19 |
| BRAFi (L+BRAFi) vs BRAFi (W3A+BRAFi) | -3.142 | 1.261 | No | ns | -12.62 to 6.335 |
| BRAFi (L+BRAFi) vs W3A (L+BRAFi) | 0.0000001788 | 0.00000007178 | No | ns | -9.477 to 9.477 |
| BRAFi (L+BRAFi) vs W3A (W3A+BRAFi) | -5.585 | 2.242 | No | ns | -15.06 to 3.892 |
| BRAFi (L+BRAFi) vs W3A+BRAFi (L+BRAFi) | -0.0000001192 | 0.00000004786 | No | ns | -9.477 to 9.477 |
| BRAFi (L+BRAFi) vs W3A+BRAFi (W3A+BRAFi) | -4.865 | 1.953 | No | ns | -14.34 to 4.612 |
| BRAFi (W3A+BRAFi) vs W3A (L+BRAFi) | 3.142 | 1.261 | No | ns | -6.335 to 12.62 |
| BRAFi (W3A+BRAFi) vs W3A (W3A+BRAFi) | -2.443 | 0.9807 | No | ns | -11.92 to 7.034 |
| BRAFi (W3A+BRAFi) vs W3A+BRAFi (L+BRAFi) | 3.142 | 1.261 | No | ns | -6.335 to 12.62 |
| BRAFi (W3A+BRAFi) vs W3A+BRAFi (W3A+BRAFi) | -1.723 | 0.6918 | No | ns | -11.20 to 7.754 |
| W3A (L+BRAFi) vs W3A (W3A+BRAFi) | -5.585 | 2.242 | No | ns | -15.06 to 3.892 |
| W3A (L+BRAFi) vs W3A+BRAFi (L+BRAFi) | -0.0000002980 | 0.0000001196 | No | ns | -9.477 to 9.477 |
| W3A (L+BRAFi) vs W3A+BRAFi (W3A+BRAFi) | -4.865 | 1.953 | No | ns | -14.34 to 4.612 |
| W3A (W3A+BRAFi) vs W3A+BRAFi (L+BRAFi) | 5.585 | 2.242 | No | ns | -3.892 to 15.06 |
| W3A (W3A+BRAFi) vs W3A+BRAFi (W3A+BRAFi) | 0.7197 | 0.2889 | No | ns | -8.757 to 10.20 |
| W3A+BRAFi (L+BRAFi) vs W3A+BRAFi (W3A+BRAFi) | -4.865 | 1.953 | No | ns | -14.34 to 4.612 |

| Table Analyzed | isolated A2058 AXIN2 |  |  |  |  |
| --- | --- | --- | --- | --- | --- |
|  |  |  |  |  |  |
| One-way analysis of variance |  |  |  |  |  |
| P value | < 0.0001 |  |  |  |  |
| P value summary | *** |  |  |  |  |
| Are means signif. different? (P < 0.05) | Yes |  |  |  |  |
| Number of groups | 10 |  |  |  |  |
| F | 50.02 |  |  |  |  |
| R squared | 0.9575 |  |  |  |  |
|  |  |  |  |  |  |
| ANOVA Table | SS | df | MS |  |  |
| Treatment (between columns) | 256.4 | 9 | 28.49 |  |  |
| Residual (within columns) | 11.39 | 20 | 0.5696 |  |  |
| Total | 267.8 | 29 |  |  |  |
|  |  |  |  |  |  |
| Bonferroni's Multiple Comparison Test | Mean Diff. | t | Significant? P < 0.05? | Summary | 95% CI of diff |
| parental (L+DMSO) vs parental (L+BRAFi) | -0.4859 | 0.7886 | No | ns | -2.830 to 1.858 |
| parental (L+DMSO) vs parental (W3A) | -5.469 | 8.876 | Yes | *** | -7.814 to -3.125 |
| parental (L+DMSO) vs parental (W3A+BRAFi) | -4.335 | 7.035 | Yes | *** | -6.679 to -1.991 |
| parental (L+DMSO) vs BRAFi (L+BRAFi) | 0.0000001192 | 0.0000001935 | No | ns | -2.344 to 2.344 |
| parental (L+DMSO) vs BRAFi (W3A+BRAFi) | -2.880 | 4.674 | Yes | ** | -5.224 to -0.5356 |
| parental (L+DMSO) vs W3A (L+BRAFi) | -0.0000002384 | 0.0000003869 | No | ns | -2.344 to 2.344 |
| parental (L+DMSO) vs W3A (W3A+BRAFi) | -8.896 | 14.44 | Yes | *** | -11.24 to -6.552 |
| parental (L+DMSO) vs W3A+BRAFi (L+BRAFi) | 0.0000001192 | 0.0000001935 | No | ns | -2.344 to 2.344 |
| parental (L+DMSO) vs W3A+BRAFi (W3A+BRAFi) | -4.336 | 7.036 | Yes | *** | -6.680 to -1.992 |
| parental (L+BRAFi) vs parental (W3A) | -4.983 | 8.087 | Yes | *** | -7.328 to -2.639 |
| parental (L+BRAFi) vs parental (W3A+BRAFi) | -3.849 | 6.246 | Yes | *** | -6.193 to -1.505 |
| parental (L+BRAFi) vs BRAFi (L+BRAFi) | 0.4859 | 0.7886 | No | ns | -1.858 to 2.830 |
| parental (L+BRAFi) vs BRAFi (W3A+BRAFi) | -2.394 | 3.885 | Yes | * | -4.738 to -0.04972 |
| parental (L+BRAFi) vs W3A (L+BRAFi) | 0.4859 | 0.7886 | No | ns | -1.858 to 2.830 |
| parental (L+BRAFi) vs W3A (W3A+BRAFi) | -8.410 | 13.65 | Yes | *** | -10.75 to -6.066 |
| parental (L+BRAFi) vs W3A+BRAFi (L+BRAFi) | 0.4859 | 0.7886 | No | ns | -1.858 to 2.830 |
| parental (L+BRAFi) vs W3A+BRAFi (W3A+BRAFi) | -3.850 | 6.248 | Yes | *** | -6.194 to -1.506 |
| parental (W3A) vs parental (W3A+BRAFi) | 1.134 | 1.841 | No | ns | -1.210 to 3.479 |
| parental (W3A) vs BRAFi (L+BRAFi) | 5.469 | 8.876 | Yes | *** | 3.125 to 7.814 |
| parental (W3A) vs BRAFi (W3A+BRAFi) | 2.589 | 4.202 | Yes | * | 0.2451 to 4.934 |
| parental (W3A) vs W3A (L+BRAFi) | 5.469 | 8.876 | Yes | *** | 3.125 to 7.814 |
| parental (W3A) vs W3A (W3A+BRAFi) | -3.427 | 5.561 | Yes | *** | -5.771 to -1.083 |
| parental (W3A) vs W3A+BRAFi (L+BRAFi) | 5.469 | 8.876 | Yes | *** | 3.125 to 7.814 |
| parental (W3A) vs W3A+BRAFi (W3A+BRAFi) | 1.133 | 1.839 | No | ns | -1.211 to 3.478 |
| parental (W3A+BRAFi) vs BRAFi (L+BRAFi) | 4.335 | 7.035 | Yes | *** | 1.991 to 6.679 |
| parental (W3A+BRAFi) vs BRAFi (W3A+BRAFi) | 1.455 | 2.361 | No | ns | -0.8893 to 3.799 |
| parental (W3A+BRAFi) vs W3A (L+BRAFi) | 4.335 | 7.035 | Yes | *** | 1.991 to 6.679 |
| parental (W3A+BRAFi) vs W3A (W3A+BRAFi) | -4.561 | 7.402 | Yes | *** | -6.906 to -2.217 |
| parental (W3A+BRAFi) vs W3A+BRAFi (L+BRAFi) | 4.335 | 7.035 | Yes | *** | 1.991 to 6.679 |
| parental (W3A+BRAFi) vs W3A+BRAFi (W3A+BRAFi) | -0.0008907 | 0.001446 | No | ns | -2.345 to 2.343 |
| BRAFi (L+BRAFi) vs BRAFi (W3A+BRAFi) | -2.880 | 4.674 | Yes | ** | -5.224 to -0.5356 |
| BRAFi (L+BRAFi) vs W3A (L+BRAFi) | -0.0000003576 | 0.0000005804 | No | ns | -2.344 to 2.344 |
| BRAFi (L+BRAFi) vs W3A (W3A+BRAFi) | -8.896 | 14.44 | Yes | *** | -11.24 to -6.552 |
| BRAFi (L+BRAFi) vs W3A+BRAFi (L+BRAFi) | 0.0000 | 0.0000 | No | ns | -2.344 to 2.344 |
| BRAFi (L+BRAFi) vs W3A+BRAFi (W3A+BRAFi) | -4.336 | 7.036 | Yes | *** | -6.680 to -1.992 |
| BRAFi (W3A+BRAFi) vs W3A (L+BRAFi) | 2.880 | 4.674 | Yes | ** | 0.5356 to 5.224 |
| BRAFi (W3A+BRAFi) vs W3A (W3A+BRAFi) | -6.016 | 9.763 | Yes | *** | -8.361 to -3.672 |
| BRAFi (W3A+BRAFi) vs W3A+BRAFi (L+BRAFi) | 2.880 | 4.674 | Yes | ** | 0.5356 to 5.224 |
| BRAFi (W3A+BRAFi) vs W3A+BRAFi (W3A+BRAFi) | -1.456 | 2.363 | No | ns | -3.800 to 0.8884 |
| W3A (L+BRAFi) vs W3A (W3A+BRAFi) | -8.896 | 14.44 | Yes | *** | -11.24 to -6.552 |
| W3A (L+BRAFi) vs W3A+BRAFi (L+BRAFi) | 0.0000003576 | 0.0000005804 | No | ns | -2.344 to 2.344 |
| W3A (L+BRAFi) vs W3A+BRAFi (W3A+BRAFi) | -4.336 | 7.036 | Yes | *** | -6.680 to -1.992 |
| W3A (W3A+BRAFi) vs W3A+BRAFi (L+BRAFi) | 8.896 | 14.44 | Yes | *** | 6.552 to 11.24 |
| W3A (W3A+BRAFi) vs W3A+BRAFi (W3A+BRAFi) | 4.560 | 7.401 | Yes | *** | 2.216 to 6.905 |
| W3A+BRAFi (L+BRAFi) vs W3A+BRAFi (W3A+BRAFi) | -4.336 | 7.036 | Yes | *** | -6.680 to -1.992 |

| Table Analyzed | isolated SKMEL28 AXIN2 |  |  |  |  |
| --- | --- | --- | --- | --- | --- |
|  |  |  |  |  |  |
| One-way analysis of variance |  |  |  |  |  |
| P value | < 0.0001 |  |  |  |  |
| P value summary | *** |  |  |  |  |
| Are means signif. different? (P < 0.05) | Yes |  |  |  |  |
| Number of groups | 10 |  |  |  |  |
| F | 22.48 |  |  |  |  |
| R squared | 0.9100 |  |  |  |  |
|  |  |  |  |  |  |
| ANOVA Table | SS | df | MS |  |  |
| Treatment (between columns) | 19.71 | 9 | 2.190 |  |  |
| Residual (within columns) | 1.949 | 20 | 0.09744 |  |  |
| Total | 21.66 | 29 |  |  |  |
|  |  |  |  |  |  |
| Bonferroni's Multiple Comparison Test | Mean Diff. | t | Significant? P < 0.05? | Summary | 95% CI of diff |
| parental (L+DMSO) vs parental (L+BRAFi) | -0.3215 | 1.261 | No | ns | -1.291 to 0.6482 |
| parental (L+DMSO) vs parental (W3A) | -1.589 | 6.233 | Yes | *** | -2.558 to -0.6191 |
| parental (L+DMSO) vs parental (W3A+BRAFi) | -2.144 | 8.411 | Yes | *** | -3.113 to -1.174 |
| parental (L+DMSO) vs BRAFi (L+BRAFi) | 0.0000001192 | 0.0000004677 | No | ns | -0.9696 to 0.9696 |
| parental (L+DMSO) vs BRAFi (W3A+BRAFi) | -0.3681 | 1.444 | No | ns | -1.338 to 0.6016 |
| parental (L+DMSO) vs W3A (L+BRAFi) | -0.0000001192 | 0.0000004677 | No | ns | -0.9696 to 0.9696 |
| parental (L+DMSO) vs W3A (W3A+BRAFi) | -1.891 | 7.418 | Yes | *** | -2.860 to -0.9209 |
| parental (L+DMSO) vs W3A+BRAFi (L+BRAFi) | 0.00000005961 | 0.0000002339 | No | ns | -0.9696 to 0.9696 |
| parental (L+DMSO) vs W3A+BRAFi (W3A+BRAFi) | -0.4325 | 1.697 | No | ns | -1.402 to 0.5371 |
| parental (L+BRAFi) vs parental (W3A) | -1.267 | 4.972 | Yes | ** | -2.237 to -0.2976 |
| parental (L+BRAFi) vs parental (W3A+BRAFi) | -1.822 | 7.150 | Yes | *** | -2.792 to -0.8527 |
| parental (L+BRAFi) vs BRAFi (L+BRAFi) | 0.3215 | 1.261 | No | ns | -0.6482 to 1.291 |
| parental (L+BRAFi) vs BRAFi (W3A+BRAFi) | -0.04661 | 0.1829 | No | ns | -1.016 to 0.9230 |
| parental (L+BRAFi) vs W3A (L+BRAFi) | 0.3215 | 1.261 | No | ns | -0.6482 to 1.291 |
| parental (L+BRAFi) vs W3A (W3A+BRAFi) | -1.569 | 6.156 | Yes | *** | -2.539 to -0.5995 |
| parental (L+BRAFi) vs W3A+BRAFi (L+BRAFi) | 0.3215 | 1.261 | No | ns | -0.6482 to 1.291 |
| parental (L+BRAFi) vs W3A+BRAFi (W3A+BRAFi) | -0.1111 | 0.4358 | No | ns | -1.081 to 0.8586 |
| parental (W3A) vs parental (W3A+BRAFi) | -0.5550 | 2.178 | No | ns | -1.525 to 0.4146 |
| parental (W3A) vs BRAFi (L+BRAFi) | 1.589 | 6.233 | Yes | *** | 0.6191 to 2.558 |
| parental (W3A) vs BRAFi (W3A+BRAFi) | 1.221 | 4.789 | Yes | ** | 0.2510 to 2.190 |
| parental (W3A) vs W3A (L+BRAFi) | 1.589 | 6.233 | Yes | *** | 0.6191 to 2.558 |
| parental (W3A) vs W3A (W3A+BRAFi) | -0.3018 | 1.184 | No | ns | -1.271 to 0.6678 |
| parental (W3A) vs W3A+BRAFi (L+BRAFi) | 1.589 | 6.233 | Yes | *** | 0.6191 to 2.558 |
| parental (W3A) vs W3A+BRAFi (W3A+BRAFi) | 1.156 | 4.536 | Yes | ** | 0.1866 to 2.126 |
| parental (W3A+BRAFi) vs BRAFi (L+BRAFi) | 2.144 | 8.411 | Yes | *** | 1.174 to 3.113 |
| parental (W3A+BRAFi) vs BRAFi (W3A+BRAFi) | 1.776 | 6.967 | Yes | *** | 0.8061 to 2.745 |
| parental (W3A+BRAFi) vs W3A (L+BRAFi) | 2.144 | 8.411 | Yes | *** | 1.174 to 3.113 |
| parental (W3A+BRAFi) vs W3A (W3A+BRAFi) | 0.2532 | 0.9935 | No | ns | -0.7164 to 1.223 |
| parental (W3A+BRAFi) vs W3A+BRAFi (L+BRAFi) | 2.144 | 8.411 | Yes | *** | 1.174 to 3.113 |
| parental (W3A+BRAFi) vs W3A+BRAFi (W3A+BRAFi) | 1.711 | 6.714 | Yes | *** | 0.7416 to 2.681 |
| BRAFi (L+BRAFi) vs BRAFi (W3A+BRAFi) | -0.3681 | 1.444 | No | ns | -1.338 to 0.6016 |
| BRAFi (L+BRAFi) vs W3A (L+BRAFi) | -0.0000002384 | 0.0000009354 | No | ns | -0.9696 to 0.9696 |
| BRAFi (L+BRAFi) vs W3A (W3A+BRAFi) | -1.891 | 7.418 | Yes | *** | -2.860 to -0.9209 |
| BRAFi (L+BRAFi) vs W3A+BRAFi (L+BRAFi) | -0.00000005961 | 0.0000002339 | No | ns | -0.9696 to 0.9696 |
| BRAFi (L+BRAFi) vs W3A+BRAFi (W3A+BRAFi) | -0.4325 | 1.697 | No | ns | -1.402 to 0.5371 |
| BRAFi (W3A+BRAFi) vs W3A (L+BRAFi) | 0.3681 | 1.444 | No | ns | -0.6016 to 1.338 |
| BRAFi (W3A+BRAFi) vs W3A (W3A+BRAFi) | -1.522 | 5.973 | Yes | *** | -2.492 to -0.5528 |
| BRAFi (W3A+BRAFi) vs W3A+BRAFi (L+BRAFi) | 0.3681 | 1.444 | No | ns | -0.6016 to 1.338 |
| BRAFi (W3A+BRAFi) vs W3A+BRAFi (W3A+BRAFi) | -0.06445 | 0.2529 | No | ns | -1.034 to 0.9052 |
| W3A (L+BRAFi) vs W3A (W3A+BRAFi) | -1.891 | 7.418 | Yes | *** | -2.860 to -0.9209 |
| W3A (L+BRAFi) vs W3A+BRAFi (L+BRAFi) | 0.0000001788 | 0.0000007016 | No | ns | -0.9696 to 0.9696 |
| W3A (L+BRAFi) vs W3A+BRAFi (W3A+BRAFi) | -0.4325 | 1.697 | No | ns | -1.402 to 0.5371 |
| W3A (W3A+BRAFi) vs W3A+BRAFi (L+BRAFi) | 1.891 | 7.418 | Yes | *** | 0.9209 to 2.860 |
| W3A (W3A+BRAFi) vs W3A+BRAFi (W3A+BRAFi) | 1.458 | 5.721 | Yes | *** | 0.4884 to 2.428 |
| W3A+BRAFi (L+BRAFi) vs W3A+BRAFi (W3A+BRAFi) | -0.4325 | 1.697 | No | ns | -1.402 to 0.5371 |

| Table Analyzed | isolated A375 cPARP |  |  |  |  |
| --- | --- | --- | --- | --- | --- |
|  |  |  |  |  |  |
| One-way analysis of variance |  |  |  |  |  |
| P value | < 0.0001 |  |  |  |  |
| P value summary | *** |  |  |  |  |
| Are means signif. different? (P < 0.05) | Yes |  |  |  |  |
| Number of groups | 10 |  |  |  |  |
| F | 565.1 |  |  |  |  |
| R squared | 0.9963 |  |  |  |  |
|  |  |  |  |  |  |
| ANOVA Table | SS | df | MS |  |  |
| Treatment (between columns) | 2648 | 9 | 294.2 |  |  |
| Residual (within columns) | 9.893 | 19 | 0.5207 |  |  |
| Total | 2658 | 28 |  |  |  |
|  |  |  |  |  |  |
| Bonferroni's Multiple Comparison Test | Mean Diff. | t | Significant? P < 0.05? | Summary | 95% CI of diff |
| parental (L+DMSO) vs parental (L+BRAFi) | -1.247 | 2.116 | No | ns | -3.507 to 1.014 |
| parental (L+DMSO) vs parental (W3A) | -1.445 | 2.194 | No | ns | -3.973 to 1.083 |
| parental (L+DMSO) vs parental (W3A+BRAFi) | -31.64 | 53.70 | Yes | *** | -33.90 to -29.38 |
| parental (L+DMSO) vs BRAFi (L+BRAFi) | -0.5900 | 1.001 | No | ns | -2.851 to 1.671 |
| parental (L+DMSO) vs BRAFi (W3A+BRAFi) | -0.2167 | 0.3677 | No | ns | -2.477 to 2.044 |
| parental (L+DMSO) vs W3A (L+BRAFi) | -0.3467 | 0.5884 | No | ns | -2.607 to 1.914 |
| parental (L+DMSO) vs W3A (W3A+BRAFi) | -4.687 | 7.955 | Yes | *** | -6.947 to -2.426 |
| parental (L+DMSO) vs W3A+BRAFi (L+BRAFi) | -9.970 | 16.92 | Yes | *** | -12.23 to -7.709 |
| parental (L+DMSO) vs W3A+BRAFi (W3A+BRAFi) | -12.54 | 21.28 | Yes | *** | -14.80 to -10.28 |
| parental (L+BRAFi) vs parental (W3A) | -0.1983 | 0.3011 | No | ns | -2.726 to 2.329 |
| parental (L+BRAFi) vs parental (W3A+BRAFi) | -30.39 | 51.58 | Yes | *** | -32.65 to -28.13 |
| parental (L+BRAFi) vs BRAFi (L+BRAFi) | 0.6567 | 1.115 | No | ns | -1.604 to 2.917 |
| parental (L+BRAFi) vs BRAFi (W3A+BRAFi) | 1.030 | 1.748 | No | ns | -1.231 to 3.291 |
| parental (L+BRAFi) vs W3A (L+BRAFi) | 0.9000 | 1.528 | No | ns | -1.361 to 3.161 |
| parental (L+BRAFi) vs W3A (W3A+BRAFi) | -3.440 | 5.839 | Yes | *** | -5.701 to -1.179 |
| parental (L+BRAFi) vs W3A+BRAFi (L+BRAFi) | -8.723 | 14.81 | Yes | *** | -10.98 to -6.463 |
| parental (L+BRAFi) vs W3A+BRAFi (W3A+BRAFi) | -11.29 | 19.16 | Yes | *** | -13.55 to -9.029 |
| parental (W3A) vs parental (W3A+BRAFi) | -30.19 | 45.83 | Yes | *** | -32.72 to -27.66 |
| parental (W3A) vs BRAFi (L+BRAFi) | 0.8550 | 1.298 | No | ns | -1.673 to 3.383 |
| parental (W3A) vs BRAFi (W3A+BRAFi) | 1.228 | 1.865 | No | ns | -1.299 to 3.756 |
| parental (W3A) vs W3A (L+BRAFi) | 1.098 | 1.667 | No | ns | -1.429 to 3.626 |
| parental (W3A) vs W3A (W3A+BRAFi) | -3.242 | 4.921 | Yes | ** | -5.769 to -0.7140 |
| parental (W3A) vs W3A+BRAFi (L+BRAFi) | -8.525 | 12.94 | Yes | *** | -11.05 to -5.997 |
| parental (W3A) vs W3A+BRAFi (W3A+BRAFi) | -11.09 | 16.84 | Yes | *** | -13.62 to -8.564 |
| parental (W3A+BRAFi) vs BRAFi (L+BRAFi) | 31.05 | 52.69 | Yes | *** | 28.79 to 33.31 |
| parental (W3A+BRAFi) vs BRAFi (W3A+BRAFi) | 31.42 | 53.33 | Yes | *** | 29.16 to 33.68 |
| parental (W3A+BRAFi) vs W3A (L+BRAFi) | 31.29 | 53.11 | Yes | *** | 29.03 to 33.55 |
| parental (W3A+BRAFi) vs W3A (W3A+BRAFi) | 26.95 | 45.74 | Yes | *** | 24.69 to 29.21 |
| parental (W3A+BRAFi) vs W3A+BRAFi (L+BRAFi) | 21.67 | 36.77 | Yes | *** | 19.41 to 23.93 |
| parental (W3A+BRAFi) vs W3A+BRAFi (W3A+BRAFi) | 19.10 | 32.42 | Yes | *** | 16.84 to 21.36 |
| BRAFi (L+BRAFi) vs BRAFi (W3A+BRAFi) | 0.3733 | 0.6337 | No | ns | -1.887 to 2.634 |
| BRAFi (L+BRAFi) vs W3A (L+BRAFi) | 0.2433 | 0.4130 | No | ns | -2.017 to 2.504 |
| BRAFi (L+BRAFi) vs W3A (W3A+BRAFi) | -4.097 | 6.953 | Yes | *** | -6.357 to -1.836 |
| BRAFi (L+BRAFi) vs W3A+BRAFi (L+BRAFi) | -9.380 | 15.92 | Yes | *** | -11.64 to -7.119 |
| BRAFi (L+BRAFi) vs W3A+BRAFi (W3A+BRAFi) | -11.95 | 20.28 | Yes | *** | -14.21 to -9.686 |
| BRAFi (W3A+BRAFi) vs W3A (L+BRAFi) | -0.1300 | 0.2206 | No | ns | -2.391 to 2.131 |
| BRAFi (W3A+BRAFi) vs W3A (W3A+BRAFi) | -4.470 | 7.587 | Yes | *** | -6.731 to -2.209 |
| BRAFi (W3A+BRAFi) vs W3A+BRAFi (L+BRAFi) | -9.753 | 16.55 | Yes | *** | -12.01 to -7.493 |
| BRAFi (W3A+BRAFi) vs W3A+BRAFi (W3A+BRAFi) | -12.32 | 20.91 | Yes | *** | -14.58 to -10.06 |
| W3A (L+BRAFi) vs W3A (W3A+BRAFi) | -4.340 | 7.366 | Yes | *** | -6.601 to -2.079 |
| W3A (L+BRAFi) vs W3A+BRAFi (L+BRAFi) | -9.623 | 16.33 | Yes | *** | -11.88 to -7.363 |
| W3A (L+BRAFi) vs W3A+BRAFi (W3A+BRAFi) | -12.19 | 20.69 | Yes | *** | -14.45 to -9.929 |
| W3A (W3A+BRAFi) vs W3A+BRAFi (L+BRAFi) | -5.283 | 8.967 | Yes | *** | -7.544 to -3.023 |
| W3A (W3A+BRAFi) vs W3A+BRAFi (W3A+BRAFi) | -7.850 | 13.32 | Yes | *** | -10.11 to -5.589 |
| W3A+BRAFi (L+BRAFi) vs W3A+BRAFi (W3A+BRAFi) | -2.567 | 4.356 | Yes | * | -4.827 to -0.3058 |

| Table Analyzed | isolated MEL624 cPARP |  |  |  |  |
| --- | --- | --- | --- | --- | --- |
|  |  |  |  |  |  |
| One-way analysis of variance |  |  |  |  |  |
| P value | < 0.0001 |  |  |  |  |
| P value summary | *** |  |  |  |  |
| Are means signif. different? (P < 0.05) | Yes |  |  |  |  |
| Number of groups | 10 |  |  |  |  |
| F | 56.15 |  |  |  |  |
| R squared | 0.9619 |  |  |  |  |
|  |  |  |  |  |  |
| ANOVA Table | SS | df | MS |  |  |
| Treatment (between columns) | 693.9 | 9 | 77.10 |  |  |
| Residual (within columns) | 27.46 | 20 | 1.373 |  |  |
| Total | 721.3 | 29 |  |  |  |
|  |  |  |  |  |  |
| Bonferroni's Multiple Comparison Test | Mean Diff. | t | Significant? P < 0.05? | Summary | 95% CI of diff |
| parental (L+DMSO) vs parental (L+BRAFi) | -2.820 | 2.948 | No | ns | -6.460 to 0.8197 |
| parental (L+DMSO) vs parental (W3A) | -0.3767 | 0.3937 | No | ns | -4.016 to 3.263 |
| parental (L+DMSO) vs parental (W3A+BRAFi) | -15.51 | 16.21 | Yes | *** | -19.15 to -11.87 |
| parental (L+DMSO) vs BRAFi (L+BRAFi) | -1.927 | 2.014 | No | ns | -5.566 to 1.713 |
| parental (L+DMSO) vs BRAFi (W3A+BRAFi) | -1.840 | 1.923 | No | ns | -5.480 to 1.800 |
| parental (L+DMSO) vs W3A (L+BRAFi) | 1.860 | 1.944 | No | ns | -1.780 to 5.500 |
| parental (L+DMSO) vs W3A (W3A+BRAFi) | -0.5800 | 0.6062 | No | ns | -4.220 to 3.060 |
| parental (L+DMSO) vs W3A+BRAFi (L+BRAFi) | 1.830 | 1.913 | No | ns | -1.810 to 5.470 |
| parental (L+DMSO) vs W3A+BRAFi (W3A+BRAFi) | 1.190 | 1.244 | No | ns | -2.450 to 4.830 |
| parental (L+BRAFi) vs parental (W3A) | 2.443 | 2.554 | No | ns | -1.196 to 6.083 |
| parental (L+BRAFi) vs parental (W3A+BRAFi) | -12.69 | 13.26 | Yes | *** | -16.33 to -9.050 |
| parental (L+BRAFi) vs BRAFi (L+BRAFi) | 0.8933 | 0.9337 | No | ns | -2.746 to 4.533 |
| parental (L+BRAFi) vs BRAFi (W3A+BRAFi) | 0.9800 | 1.024 | No | ns | -2.660 to 4.620 |
| parental (L+BRAFi) vs W3A (L+BRAFi) | 4.680 | 4.892 | Yes | ** | 1.040 to 8.320 |
| parental (L+BRAFi) vs W3A (W3A+BRAFi) | 2.240 | 2.341 | No | ns | -1.400 to 5.880 |
| parental (L+BRAFi) vs W3A+BRAFi (L+BRAFi) | 4.650 | 4.860 | Yes | ** | 1.010 to 8.290 |
| parental (L+BRAFi) vs W3A+BRAFi (W3A+BRAFi) | 4.010 | 4.191 | Yes | * | 0.3703 to 7.650 |
| parental (W3A) vs parental (W3A+BRAFi) | -15.13 | 15.82 | Yes | *** | -18.77 to -11.49 |
| parental (W3A) vs BRAFi (L+BRAFi) | -1.550 | 1.620 | No | ns | -5.190 to 2.090 |
| parental (W3A) vs BRAFi (W3A+BRAFi) | -1.463 | 1.530 | No | ns | -5.103 to 2.176 |
| parental (W3A) vs W3A (L+BRAFi) | 2.237 | 2.338 | No | ns | -1.403 to 5.876 |
| parental (W3A) vs W3A (W3A+BRAFi) | -0.2033 | 0.2125 | No | ns | -3.843 to 3.436 |
| parental (W3A) vs W3A+BRAFi (L+BRAFi) | 2.207 | 2.306 | No | ns | -1.433 to 5.846 |
| parental (W3A) vs W3A+BRAFi (W3A+BRAFi) | 1.567 | 1.638 | No | ns | -2.073 to 5.206 |
| parental (W3A+BRAFi) vs BRAFi (L+BRAFi) | 13.58 | 14.20 | Yes | *** | 9.944 to 17.22 |
| parental (W3A+BRAFi) vs BRAFi (W3A+BRAFi) | 13.67 | 14.29 | Yes | *** | 10.03 to 17.31 |
| parental (W3A+BRAFi) vs W3A (L+BRAFi) | 17.37 | 18.16 | Yes | *** | 13.73 to 21.01 |
| parental (W3A+BRAFi) vs W3A (W3A+BRAFi) | 14.93 | 15.61 | Yes | *** | 11.29 to 18.57 |
| parental (W3A+BRAFi) vs W3A+BRAFi (L+BRAFi) | 17.34 | 18.12 | Yes | *** | 13.70 to 20.98 |
| parental (W3A+BRAFi) vs W3A+BRAFi (W3A+BRAFi) | 16.70 | 17.46 | Yes | *** | 13.06 to 20.34 |
| BRAFi (L+BRAFi) vs BRAFi (W3A+BRAFi) | 0.08667 | 0.09059 | No | ns | -3.553 to 3.726 |
| BRAFi (L+BRAFi) vs W3A (L+BRAFi) | 3.787 | 3.958 | Yes | * | 0.1469 to 7.426 |
| BRAFi (L+BRAFi) vs W3A (W3A+BRAFi) | 1.347 | 1.408 | No | ns | -2.293 to 4.986 |
| BRAFi (L+BRAFi) vs W3A+BRAFi (L+BRAFi) | 3.757 | 3.927 | Yes | * | 0.1169 to 7.396 |
| BRAFi (L+BRAFi) vs W3A+BRAFi (W3A+BRAFi) | 3.117 | 3.258 | No | ns | -0.5231 to 6.756 |
| BRAFi (W3A+BRAFi) vs W3A (L+BRAFi) | 3.700 | 3.867 | Yes | * | 0.06026 to 7.340 |
| BRAFi (W3A+BRAFi) vs W3A (W3A+BRAFi) | 1.260 | 1.317 | No | ns | -2.380 to 4.900 |
| BRAFi (W3A+BRAFi) vs W3A+BRAFi (L+BRAFi) | 3.670 | 3.836 | Yes | * | 0.03026 to 7.310 |
| BRAFi (W3A+BRAFi) vs W3A+BRAFi (W3A+BRAFi) | 3.030 | 3.167 | No | ns | -0.6097 to 6.670 |
| W3A (L+BRAFi) vs W3A (W3A+BRAFi) | -2.440 | 2.550 | No | ns | -6.080 to 1.200 |
| W3A (L+BRAFi) vs W3A+BRAFi (L+BRAFi) | -0.03000 | 0.03136 | No | ns | -3.670 to 3.610 |
| W3A (L+BRAFi) vs W3A+BRAFi (W3A+BRAFi) | -0.6700 | 0.7003 | No | ns | -4.310 to 2.970 |
| W3A (W3A+BRAFi) vs W3A+BRAFi (L+BRAFi) | 2.410 | 2.519 | No | ns | -1.230 to 6.050 |
| W3A (W3A+BRAFi) vs W3A+BRAFi (W3A+BRAFi) | 1.770 | 1.850 | No | ns | -1.870 to 5.410 |
| W3A+BRAFi (L+BRAFi) vs W3A+BRAFi (W3A+BRAFi) | -0.6400 | 0.6689 | No | ns | -4.280 to 3.000 |

| Table Analyzed | isolated A2058 cPARP |  |  |  |  |
| --- | --- | --- | --- | --- | --- |
|  |  |  |  |  |  |
| One-way analysis of variance |  |  |  |  |  |
| P value | < 0.0001 |  |  |  |  |
| P value summary | *** |  |  |  |  |
| Are means signif. different? (P < 0.05) | Yes |  |  |  |  |
| Number of groups | 10 |  |  |  |  |
| F | 9.256 |  |  |  |  |
| R squared | 0.8064 |  |  |  |  |
|  |  |  |  |  |  |
| ANOVA Table | SS | df | MS |  |  |
| Treatment (between columns) | 19.09 | 9 | 2.121 |  |  |
| Residual (within columns) | 4.584 | 20 | 0.2292 |  |  |
| Total | 23.67 | 29 |  |  |  |
|  |  |  |  |  |  |
| Bonferroni's Multiple Comparison Test | Mean Diff. | t | Significant? P < 0.05? | Summary | 95% CI of diff |
| parental (L+DMSO) vs parental (L+BRAFi) | -0.6533 | 1.671 | No | ns | -2.140 to 0.8337 |
| parental (L+DMSO) vs parental (W3A) | 0.04000 | 0.1023 | No | ns | -1.447 to 1.527 |
| parental (L+DMSO) vs parental (W3A+BRAFi) | -1.453 | 3.718 | No | ns | -2.940 to 0.03370 |
| parental (L+DMSO) vs BRAFi (L+BRAFi) | -1.570 | 4.017 | Yes | * | -3.057 to -0.08297 |
| parental (L+DMSO) vs BRAFi (W3A+BRAFi) | -1.800 | 4.605 | Yes | ** | -3.287 to -0.3130 |
| parental (L+DMSO) vs W3A (L+BRAFi) | 0.2700 | 0.6908 | No | ns | -1.217 to 1.757 |
| parental (L+DMSO) vs W3A (W3A+BRAFi) | -0.8667 | 2.217 | No | ns | -2.354 to 0.6204 |
| parental (L+DMSO) vs W3A+BRAFi (L+BRAFi) | -2.063 | 5.279 | Yes | ** | -3.550 to -0.5763 |
| parental (L+DMSO) vs W3A+BRAFi (W3A+BRAFi) | -1.497 | 3.829 | Yes | * | -2.984 to -0.009635 |
| parental (L+BRAFi) vs parental (W3A) | 0.6933 | 1.774 | No | ns | -0.7937 to 2.180 |
| parental (L+BRAFi) vs parental (W3A+BRAFi) | -0.8000 | 2.047 | No | ns | -2.287 to 0.6870 |
| parental (L+BRAFi) vs BRAFi (L+BRAFi) | -0.9167 | 2.345 | No | ns | -2.404 to 0.5704 |
| parental (L+BRAFi) vs BRAFi (W3A+BRAFi) | -1.147 | 2.934 | No | ns | -2.634 to 0.3404 |
| parental (L+BRAFi) vs W3A (L+BRAFi) | 0.9233 | 2.362 | No | ns | -0.5637 to 2.410 |
| parental (L+BRAFi) vs W3A (W3A+BRAFi) | -0.2133 | 0.5458 | No | ns | -1.700 to 1.274 |
| parental (L+BRAFi) vs W3A+BRAFi (L+BRAFi) | -1.410 | 3.607 | No | ns | -2.897 to 0.07703 |
| parental (L+BRAFi) vs W3A+BRAFi (W3A+BRAFi) | -0.8433 | 2.158 | No | ns | -2.330 to 0.6437 |
| parental (W3A) vs parental (W3A+BRAFi) | -1.493 | 3.820 | Yes | * | -2.980 to -0.006302 |
| parental (W3A) vs BRAFi (L+BRAFi) | -1.610 | 4.119 | Yes | * | -3.097 to -0.1230 |
| parental (W3A) vs BRAFi (W3A+BRAFi) | -1.840 | 4.707 | Yes | ** | -3.327 to -0.3530 |
| parental (W3A) vs W3A (L+BRAFi) | 0.2300 | 0.5884 | No | ns | -1.257 to 1.717 |
| parental (W3A) vs W3A (W3A+BRAFi) | -0.9067 | 2.320 | No | ns | -2.394 to 0.5804 |
| parental (W3A) vs W3A+BRAFi (L+BRAFi) | -2.103 | 5.381 | Yes | ** | -3.590 to -0.6163 |
| parental (W3A) vs W3A+BRAFi (W3A+BRAFi) | -1.537 | 3.931 | Yes | * | -3.024 to -0.04963 |
| parental (W3A+BRAFi) vs BRAFi (L+BRAFi) | -0.1167 | 0.2985 | No | ns | -1.604 to 1.370 |
| parental (W3A+BRAFi) vs BRAFi (W3A+BRAFi) | -0.3467 | 0.8869 | No | ns | -1.834 to 1.140 |
| parental (W3A+BRAFi) vs W3A (L+BRAFi) | 1.723 | 4.409 | Yes | * | 0.2363 to 3.210 |
| parental (W3A+BRAFi) vs W3A (W3A+BRAFi) | 0.5867 | 1.501 | No | ns | -0.9004 to 2.074 |
| parental (W3A+BRAFi) vs W3A+BRAFi (L+BRAFi) | -0.6100 | 1.561 | No | ns | -2.097 to 0.8770 |
| parental (W3A+BRAFi) vs W3A+BRAFi (W3A+BRAFi) | -0.04333 | 0.1109 | No | ns | -1.530 to 1.444 |
| BRAFi (L+BRAFi) vs BRAFi (W3A+BRAFi) | -0.2300 | 0.5884 | No | ns | -1.717 to 1.257 |
| BRAFi (L+BRAFi) vs W3A (L+BRAFi) | 1.840 | 4.707 | Yes | ** | 0.3530 to 3.327 |
| BRAFi (L+BRAFi) vs W3A (W3A+BRAFi) | 0.7033 | 1.799 | No | ns | -0.7837 to 2.190 |
| BRAFi (L+BRAFi) vs W3A+BRAFi (L+BRAFi) | -0.4933 | 1.262 | No | ns | -1.980 to 0.9937 |
| BRAFi (L+BRAFi) vs W3A+BRAFi (W3A+BRAFi) | 0.07333 | 0.1876 | No | ns | -1.414 to 1.560 |
| BRAFi (W3A+BRAFi) vs W3A (L+BRAFi) | 2.070 | 5.296 | Yes | ** | 0.5830 to 3.557 |
| BRAFi (W3A+BRAFi) vs W3A (W3A+BRAFi) | 0.9333 | 2.388 | No | ns | -0.5537 to 2.420 |
| BRAFi (W3A+BRAFi) vs W3A+BRAFi (L+BRAFi) | -0.2633 | 0.6737 | No | ns | -1.750 to 1.224 |
| BRAFi (W3A+BRAFi) vs W3A+BRAFi (W3A+BRAFi) | 0.3033 | 0.7760 | No | ns | -1.184 to 1.790 |
| W3A (L+BRAFi) vs W3A (W3A+BRAFi) | -1.137 | 2.908 | No | ns | -2.624 to 0.3504 |
| W3A (L+BRAFi) vs W3A+BRAFi (L+BRAFi) | -2.333 | 5.969 | Yes | *** | -3.820 to -0.8463 |
| W3A (L+BRAFi) vs W3A+BRAFi (W3A+BRAFi) | -1.767 | 4.520 | Yes | ** | -3.254 to -0.2796 |
| W3A (W3A+BRAFi) vs W3A+BRAFi (L+BRAFi) | -1.197 | 3.061 | No | ns | -2.684 to 0.2904 |
| W3A (W3A+BRAFi) vs W3A+BRAFi (W3A+BRAFi) | -0.6300 | 1.612 | No | ns | -2.117 to 0.8570 |
| W3A+BRAFi (L+BRAFi) vs W3A+BRAFi (W3A+BRAFi) | 0.5667 | 1.450 | No | ns | -0.9204 to 2.054 |

| Table Analyzed | isolated SKMEL28 cPARP |  |  |  |  |
| --- | --- | --- | --- | --- | --- |
|  |  |  |  |  |  |
| One-way analysis of variance |  |  |  |  |  |
| P value | < 0.0001 |  |  |  |  |
| P value summary | *** |  |  |  |  |
| Are means signif. different? (P < 0.05) | Yes |  |  |  |  |
| Number of groups | 10 |  |  |  |  |
| F | 30.61 |  |  |  |  |
| R squared | 0.9323 |  |  |  |  |
|  |  |  |  |  |  |
| ANOVA Table | SS | df | MS |  |  |
| Treatment (between columns) | 101.0 | 9 | 11.23 |  |  |
| Residual (within columns) | 7.336 | 20 | 0.3668 |  |  |
| Total | 108.4 | 29 |  |  |  |
|  |  |  |  |  |  |
| Bonferroni's Multiple Comparison Test | Mean Diff. | t | Significant? P < 0.05? | Summary | 95% CI of diff |
| parental (L+DMSO) vs parental (L+BRAFi) | -0.3400 | 0.6876 | No | ns | -2.221 to 1.541 |
| parental (L+DMSO) vs parental (W3A) | 0.06333 | 0.1281 | No | ns | -1.818 to 1.945 |
| parental (L+DMSO) vs parental (W3A+BRAFi) | -1.330 | 2.690 | No | ns | -3.211 to 0.5512 |
| parental (L+DMSO) vs BRAFi (L+BRAFi) | -4.130 | 8.352 | Yes | *** | -6.011 to -2.249 |
| parental (L+DMSO) vs BRAFi (W3A+BRAFi) | -5.697 | 11.52 | Yes | *** | -7.578 to -3.815 |
| parental (L+DMSO) vs W3A (L+BRAFi) | -0.3400 | 0.6876 | No | ns | -2.221 to 1.541 |
| parental (L+DMSO) vs W3A (W3A+BRAFi) | -2.330 | 4.712 | Yes | ** | -4.211 to -0.4488 |
| parental (L+DMSO) vs W3A+BRAFi (L+BRAFi) | -0.7300 | 1.476 | No | ns | -2.611 to 1.151 |
| parental (L+DMSO) vs W3A+BRAFi (W3A+BRAFi) | -0.7967 | 1.611 | No | ns | -2.678 to 1.085 |
| parental (L+BRAFi) vs parental (W3A) | 0.4033 | 0.8157 | No | ns | -1.478 to 2.285 |
| parental (L+BRAFi) vs parental (W3A+BRAFi) | -0.9900 | 2.002 | No | ns | -2.871 to 0.8912 |
| parental (L+BRAFi) vs BRAFi (L+BRAFi) | -3.790 | 7.664 | Yes | *** | -5.671 to -1.909 |
| parental (L+BRAFi) vs BRAFi (W3A+BRAFi) | -5.357 | 10.83 | Yes | *** | -7.238 to -3.475 |
| parental (L+BRAFi) vs W3A (L+BRAFi) | 0.0000 | 0.0000 | No | ns | -1.881 to 1.881 |
| parental (L+BRAFi) vs W3A (W3A+BRAFi) | -1.990 | 4.024 | Yes | * | -3.871 to -0.1088 |
| parental (L+BRAFi) vs W3A+BRAFi (L+BRAFi) | -0.3900 | 0.7887 | No | ns | -2.271 to 1.491 |
| parental (L+BRAFi) vs W3A+BRAFi (W3A+BRAFi) | -0.4567 | 0.9235 | No | ns | -2.338 to 1.425 |
| parental (W3A) vs parental (W3A+BRAFi) | -1.393 | 2.818 | No | ns | -3.275 to 0.4879 |
| parental (W3A) vs BRAFi (L+BRAFi) | -4.193 | 8.480 | Yes | *** | -6.075 to -2.312 |
| parental (W3A) vs BRAFi (W3A+BRAFi) | -5.760 | 11.65 | Yes | *** | -7.641 to -3.879 |
| parental (W3A) vs W3A (L+BRAFi) | -0.4033 | 0.8157 | No | ns | -2.285 to 1.478 |
| parental (W3A) vs W3A (W3A+BRAFi) | -2.393 | 4.840 | Yes | ** | -4.275 to -0.5121 |
| parental (W3A) vs W3A+BRAFi (L+BRAFi) | -0.7933 | 1.604 | No | ns | -2.675 to 1.088 |
| parental (W3A) vs W3A+BRAFi (W3A+BRAFi) | -0.8600 | 1.739 | No | ns | -2.741 to 1.021 |
| parental (W3A+BRAFi) vs BRAFi (L+BRAFi) | -2.800 | 5.662 | Yes | *** | -4.681 to -0.9188 |
| parental (W3A+BRAFi) vs BRAFi (W3A+BRAFi) | -4.367 | 8.831 | Yes | *** | -6.248 to -2.485 |
| parental (W3A+BRAFi) vs W3A (L+BRAFi) | 0.9900 | 2.002 | No | ns | -0.8912 to 2.871 |
| parental (W3A+BRAFi) vs W3A (W3A+BRAFi) | -1.000 | 2.022 | No | ns | -2.881 to 0.8812 |
| parental (W3A+BRAFi) vs W3A+BRAFi (L+BRAFi) | 0.6000 | 1.213 | No | ns | -1.281 to 2.481 |
| parental (W3A+BRAFi) vs W3A+BRAFi (W3A+BRAFi) | 0.5333 | 1.079 | No | ns | -1.348 to 2.415 |
| BRAFi (L+BRAFi) vs BRAFi (W3A+BRAFi) | -1.567 | 3.168 | No | ns | -3.448 to 0.3145 |
| BRAFi (L+BRAFi) vs W3A (L+BRAFi) | 3.790 | 7.664 | Yes | *** | 1.909 to 5.671 |
| BRAFi (L+BRAFi) vs W3A (W3A+BRAFi) | 1.800 | 3.640 | No | ns | -0.08121 to 3.681 |
| BRAFi (L+BRAFi) vs W3A+BRAFi (L+BRAFi) | 3.400 | 6.876 | Yes | *** | 1.519 to 5.281 |
| BRAFi (L+BRAFi) vs W3A+BRAFi (W3A+BRAFi) | 3.333 | 6.741 | Yes | *** | 1.452 to 5.215 |
| BRAFi (W3A+BRAFi) vs W3A (L+BRAFi) | 5.357 | 10.83 | Yes | *** | 3.475 to 7.238 |
| BRAFi (W3A+BRAFi) vs W3A (W3A+BRAFi) | 3.367 | 6.808 | Yes | *** | 1.485 to 5.248 |
| BRAFi (W3A+BRAFi) vs W3A+BRAFi (L+BRAFi) | 4.967 | 10.04 | Yes | *** | 3.085 to 6.848 |
| BRAFi (W3A+BRAFi) vs W3A+BRAFi (W3A+BRAFi) | 4.900 | 9.909 | Yes | *** | 3.019 to 6.781 |
| W3A (L+BRAFi) vs W3A (W3A+BRAFi) | -1.990 | 4.024 | Yes | * | -3.871 to -0.1088 |
| W3A (L+BRAFi) vs W3A+BRAFi (L+BRAFi) | -0.3900 | 0.7887 | No | ns | -2.271 to 1.491 |
| W3A (L+BRAFi) vs W3A+BRAFi (W3A+BRAFi) | -0.4567 | 0.9235 | No | ns | -2.338 to 1.425 |
| W3A (W3A+BRAFi) vs W3A+BRAFi (L+BRAFi) | 1.600 | 3.236 | No | ns | -0.2812 to 3.481 |
| W3A (W3A+BRAFi) vs W3A+BRAFi (W3A+BRAFi) | 1.533 | 3.101 | No | ns | -0.3479 to 3.415 |
| W3A+BRAFi (L+BRAFi) vs W3A+BRAFi (W3A+BRAFi) | -0.06667 | 0.1348 | No | ns | -1.948 to 1.815 |
